# Design and Validation of Hybrid Polymer‐Lipid Nanoparticles as Novel Transfection Vectors for MicroRNA Delivery to Human Cardiac Fibroblasts

**DOI:** 10.1002/adhm.202500971

**Published:** 2025-06-06

**Authors:** Letizia Nicoletti, Camilla Paoletti, Martina Coletto, Elena Marcello, Giovanni Paolo Stola, Francesca Cossetta, Francesco Schiavone, Ilaria Andreana, Barbara Stella, Silvia Arpicco, Clara Mattu, Valeria Chiono

**Affiliations:** ^1^ Department of Mechanical and Aerospace Engineering Politecnico di Torino Corso Duca degli Abruzzi 24 Turin 10129 Italy; ^2^ PoliRNA Srl Via Vincenzo Vela 42 Turin 10128 Italy; ^3^ Department of Drug Science and Technology University of Turin Via Pietro Giuria, 9 Turin 10125 Italy

**Keywords:** human cardiac fibroblasts, hybrid nanoparticles, microRNA, nanoparticles storage, transfection vectors

## Abstract

Hybrid polymer‐lipid nanoparticles (hybrid NPs) are developed as novel in vitro transfection vectors for microRNAs (miRNAs) delivery to overcome the poor stability, incomplete loading efficiency and fast release kinetics of commercial transfection agents. Hybrid NPs with nanometric size are prepared by a scalable high‐yield nanoprecipitation method. They consisted of a lipoplex core, composed of the cationic lipid [2‐(2,3‐didodecyloxypropyl)‐hydroxyethyl] ammonium bromide (DE) and helper lipid dioleoyl phosphatidylethanolamine (DOPE), providing 99% miRNA loading, and a poly(lactic acid‐*co*‐glycolic acid) (PLGA) shell, ensuring NPs colloidal stability and controlled miRNA release kinetics. Adult human cardiac fibroblasts (AHCFs), transiently transfected with miR‐1 loaded hybrid NPs versus RNAiMAX showed superior viability and higher miRNA content over 48 h. Hybrid NPs could be stored up to 14 days at −20 °C, upon freeze‐drying with trehalose cryoprotectant (12% w/v), regaining their physicochemical and biological properties when redispersed. Hybrid NPs are assessed in a miRcombo model of fibroblast‐to‐cardiomyocyte reprogramming. At 15 days post‐transfection with reprogramming miRNAs (miRcombo: miRs‐1, 133, 208 and 499), cardiac troponin T marker expression is significantly increased at gene and protein level. These results pave the way to hybrid NP use as transfection vectors for the in vitro testing of miRNAs targeting AHCFs.

## Graphical abstract

Hybrid NPs are developed as efficient, scalable nanocarriers for in vitro microRNA delivery, ensuring high loading efficiency, controlled release, physiological and long‐term storage stability. They outperform commercial lipid‐based reagents in terms of transfection efficiency and biocompatibility. Hybrid NPs represent a promising platform for cardiac regenerative medicine, capable of inducing direct cell reprogramming.

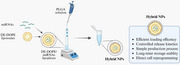

## 1 Introduction

MicroRNA (miRNAs) are small non‐coding RNAs (21–24 nucleotides in lengths) that regulate gene expression at the post‐transcriptional level,^[^ ^1^ ^]^ in several physiological and pathological processes, including cancer development and tissue regeneration.^[^ ^2^, ^3^, ^4^, ^5^, ^6^, ^7^, ^8^, ^9^, ^10^ ^]^ The expression of endogenous miRNAs can be enhanced by double‐stranded miRNA mimics, or inhibited by single‐stranded anti‐miRNA oligonucleotides (AMOs).^[^ ^4^ ^]^ In vitro and in vivo preclinical studies on new miRNA‐based therapies have been mainly performed using viral vectors, such as adeno‐associated (AAVs), retroviral (RVs), and lentiviral vectors (LVs).^[^ ^8^, ^11^, ^12^, ^13^, ^14^ ^]^ Despite their high transfection efficiency, viral vectors are limited by safety issues and high cost. Preclinical research on new miRNA therapies could benefit from safe and efficient miRNA‐loaded nanocarriers, able to protect miRNAs from degradation and enhance their uptake by cells.^[^ ^8^, ^15^, ^16^ ^]^ So far, most transfection reagents have been based on cationic lipids or polymers, able to self‐assemble into nanocomplexes in the presence of negatively charged miRNAs, forming lipoplexes or polyplexes, respectively.^[^ ^17^, ^18^, ^19^, ^20^, ^21^, ^22^, ^23^, ^24^, ^25^ ^]^ A variety of cationic lipids, such as [2‐(2,3‐didodecyloxypropyl)‐hydroxyethyl] ammonium bromide (DE),^[^ ^5^, ^26^, ^27^, ^28^, ^29^, ^30^ ^]^ N‐[1‐(2,3‐dioleyloxy) propyl‐N,N,N‐trimethylammonium chloride (DOTMA) and 1,2‐dioleoyl‐3‐trimethylammonium‐propane (DOTAP) have been used to form lipoplexes.^[^ ^31^, ^32^ ^]^ The positive charge of quaternary amino groups of cationic lipids is responsible for their electrostatic interactions with negatively charged nucleic acids, leading to lipoplexes formation with high loading efficiency.^[^ ^31^ ^]^ Examples include lipoplexes formed from commercial Lipofectamine and its derivatives (e.g., Lipofectamine 3000, Lipofectamine 2000, Lipofectamine RNAiMAX, and Lipofectin).^[^ ^17^, ^18^, ^19^, ^20^, ^21^, ^23^, ^25^, ^33^ ^]^ Among these, Lipofectamine RNAiMAX is widely used for the delivery of miRNAs and small interfering RNAs (siRNA) to different cells, including cardiomyocytes (CMs).^[^ ^13^, ^20^, ^21^, ^23^ ^]^ Other commercially available reagents based on cationic lipids for in vitro miRNA delivery, include DharmaFECT (Dharmacon),^[^ ^2^, ^5^, ^7^, ^34^, ^35^ ^]^ SiPORT (Invitrogen),^[^ ^36^ ^]^ SilentFect (Bio‐Rad)^[^ ^37^ ^]^ and INTERFERin (Polyplus).^[^ ^38^, ^39^, ^40^, ^41^, ^42^ ^]^ These commercial transfection reagents have demonstrated high loading and transfection efficiency which varies depending on cell types. However, cationic lipoplexes are limited by their high average size (often > 1 µm) and wide size distribution, cytotoxicity, and low stability in physiological media, potentially leading to miRNA release before cell internalization.^[^ ^13^, ^17^, ^19^ ^]^ Transfection reagents based on cationic polymers, such as poly(ethyleneimine) (PEI) and chitosan, have been proposed as an alternative to cationic lipids to produce polyplexes.^[^ ^24^, ^25^, ^31^, ^43^ ^]^ PEI polyplexes have been shown to efficiently encapsulate oligonucleotides and to release them efficiently in the cell cytoplasm by endosomal escape mediated by the proton sponge effect.^[^ ^31^ ^]^ One example of commercially available PEI transfection reagents is jetPEI from Polyplus.^[^ ^24^ ^]^ However, PEI polyplexes are limited by non‐degradability and cytotoxicity.^[^ ^32^ ^]^ Chitosan has also been used to prepare miRNA‐loaded nanoparticles (NPs), for its positive charge at slightly acidic pH (< pKa ≈6.3), biocompatibility, and biodegradability.^[^ ^31^, ^43^, ^44^ ^]^ However, the preparation of chitosan polyplexes at acidic pH may cause partial miRNA degradation and reduce the stability of polyplexes in the blood. In addition to hydrophilic cationic polymers, poly(lactic acid‐*co*‐glycolic acid) (PLGA) has been widely used for drug delivery due to its stability in physiological media, biodegradability, biocompatibility, and ability of endosomal escape.^[^ ^45^, ^46^ ^]^ However, PLGA NPs suffer from poor loading of hydrophilic molecules, such as oligonucleotides (e.g., miRNA and siRNA).^[^ ^47^ ^]^ To improve oligonucleotide loading, hybrid NPs based on PLGA and cationic polymers or lipids have been proposed.^[^ ^43^, ^48^, ^49^, ^50^, ^51^, ^52^, ^53^, ^54^ ^]^ PLGA NPs coated with cationic polymers (e.g., PEI or chitosan),^[^ ^44^, ^48^, ^49^, ^50^, ^52^, ^53^ ^]^ or lipids (e.g., DOTAP or DOTMA)^[^ ^44^, ^55^ ^]^ have been prepared by oil‐in‐water (o/w) single emulsion method or by nanoprecipitation. Transfection reagents based on PEI‐coated PLGA NPs are also commercially available (DiagPoly PEI PLGA), ready for complexation with oligonucleotides by users. However, PLGA NPs coated with cationic polymers or lipids suffer from limited stability in physiological conditions and cytotoxicity issues, due to their positive surface charge.^[^ ^46^ ^]^ Alternatively, PLGA NPs encapsulating lipoplexes or polyplexes have been prepared by water‐in‐oil‐in‐water (w/o/w) double emulsion methods.^[^ ^46^ ^]^ These particles possess a lipoplex or polyplex core, which provides high loading efficiency of miRNAs, while the PLGA shell confers stability in physiological conditions, and controlled and sustained release of the payload.^[^ ^45^ ^]^ Unfortunately, the inherent limitations of the double‐emulsion method, including complexity, time demand, low yield, use of potentially toxic surfactants, and poor encapsulation efficiency, have limited the applicability of these NPs as transfection reagents for wide research applications.^[^ ^55^ ^]^ To overcome such limitations, we designed novel hybrid NPs with a lipoplex core and a PLGA shell, by a simple, user‐friendly, high‐yield, and scalable nanoprecipitation method, proposing their use as novel miRNA transfection vectors for research use, with a focus on their application for cardiac regenerative medicine.^[^ ^45^ ^]^ miRNA‐loaded lipoplexes based on a cationic lipid (DE) and a helper lipid (dioleoyl phosphatidylethanolamine, DOPE) were first prepared by simple electrostatic interaction in water. In our previous work, DE‐DOPE/miRNA lipoplexes were found to have ≈100% miRNA loading efficiency (LE), a fast and complete miRNA release within 24 h, high transfection ability, and biocompatibility, and to trigger efficient adult human cardiac fibroblasts (AHCFs) reprogramming into induced CMs when delivering miRcombo (iCMs).^[^ ^5^, ^10^ ^]^ Herein, a PLGA solution was nano‐precipitated on a dispersion of DE‐DOPE/miRNA lipoplexes,^[^ ^45^ ^]^ obtaining hybrid NPs The resulting hybrid NPs combined the high miRNA loading of DE‐DOPE/miRNA lipoplexes with the structural stability and controlled release ability of PLGA NPs.^[^ ^5^, ^6^, ^7^, ^8^, ^9^, ^10^, ^15^, ^16^, ^45^ ^]^ Hybrid NPs were thoroughly characterized for their physicochemical and biological properties for miRNA delivery to AHCFs, compared to Lipofectamine RNAiMAX (RNAiMAX), one of the most relevant miRNA and siRNA commercial lipid transfection agents. Proof of concept application of hybrid NPs for AHCFs transfection was demonstrated using a single miRNA (miR‐1) and a reprogramming miRNA combination (miRcombo: miR‐1‐3p, miR‐133a‐3p, miR‐208a‐3p, miR‐499a‐5p). Results demonstrated efficient miRcombo‐mediated direct reprogramming of AHCFs toward iCMs providing a reliable proof‐of‐concept for the application of hybrid NPs as transfection reagents for research use.

## 2 Results

### 2.1 Physicochemical Properties of Hybrid NPs

**Figure 1:**
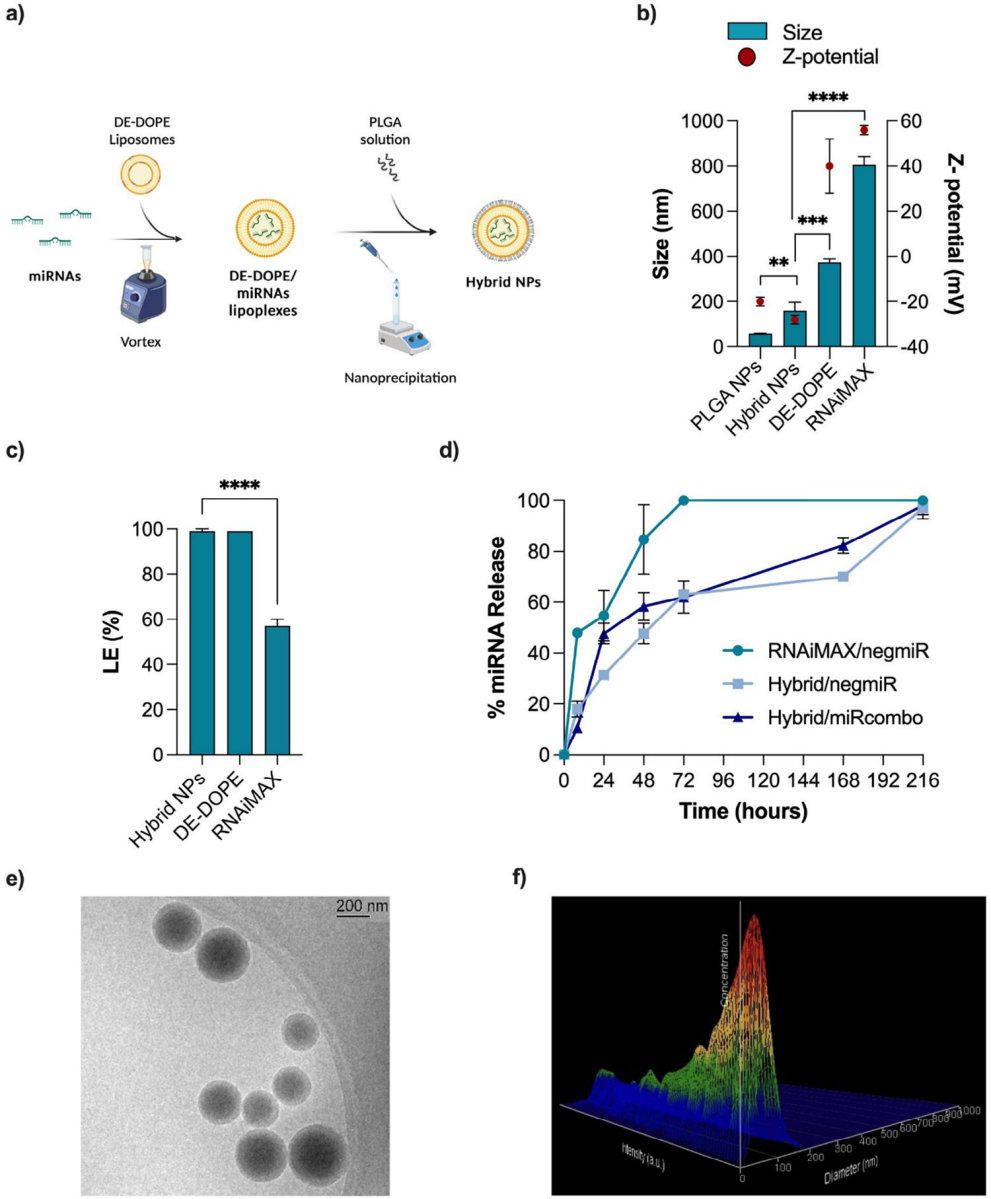
Physicochemical properties of hybrid NPs: a) Schematic illustration of the nanoprecipitation protocol for the preparation of hybrid NPs. Created with Biorender. b) Size and Z‐potential of control PLGA NPs (PLGA NPs), hybrid NPs, and RNAiMAX, measured by DLS. NegmiR was loaded as model miRNA. c) NegmiR loading efficiency (LE) measured by Qubit fluorometric quantification assay for hybrid NPs compared to RNAiMAX. d) Release profile of negmiR and miRcombo from hybrid NPs and of negmiR from RNAiMAX NPs as a function of their incubation time (24, 48, 72, and 168 h) in PBS at 37 °C under dynamic conditions. Data are expressed as mean ± SD. NegmiR was loaded as model miRNA. Statistical analysis was performed by 1‐way ANOVA. e) Representative Cryo‐TEM images of hybrid NPs. f) 3D topographic image of hybrid NPs by NTA analysis. Data are expressed as mean ± SD. Statistical analysis was performed by a two‐sided *t*‐test.

MiRNA‐loaded hybrid NPs were prepared by a one‐step nanoprecipitation approach, adapted from a previously reported protocol.^[^ ^52^ ^]^ As schematically illustrated in **Figure** **1**a and reported in Figure S1 (Supporting Information), different amounts of PLGA solution in acetone (16–127 µg range) were dropped into an aqueous phase consisting of a dispersion of previously optimized DE‐DOPE/miRNA lipoplexes^[^ ^5^ ^]^ (amino to phosphate ratio – N/P – of 3). By increasing the PLGA content (% w/w) in the hybrid NPs from 70 to 95% w/w, the average hydrodynamic diameter, Z‐potential, and polydispersity index (PDI) of resulting hybrid NPs decreased (Figure S1 and Table S1, Supporting information). Particularly, only hybrid NPs with a PLGA content > 80% showed a hydrodynamic size < 200 nm which is a threshold value allowing sterilization by mechanical filtration. Within this range of composition, hybrid NPs with a PLGA content of 95% w/w in the resulting hybrid NPs were selected for further characterizations, considering their lowest hydrodynamic size (160 ± 36 nm), combined with a negative Z‐potential (−28 ± 2 mV), relatively low PDI (0.27 ± 0.05) (Figure S1 and Table S1, Supporting Information) and high loading efficiency (LE) percentage (99 ± 0.01%). Different types of oligonucleotides (control negmiR, miR‐1, miRcombo, Cy5‐siRNA) were loaded into the hybrid NPs for characterization purposes.

Physicochemical properties of hybrid NPs were analyzed and compared to control DE‐DOPE/miRNA lipoplexes^[^ ^5^ ^]^ and RNAiMAX NPs loaded with negmiR and PLGA NPs, non‐encapsulating miRNA (Figure 1b; Table S2, Supporting Information). The hydrodynamic diameter of negmiR‐loaded hybrid NPs was higher than for PLGA NPs (160 ± 36 nm vs 59 ± 1 nm, p = 0.0083), while lower than that of DE‐DOPE/miRNA lipoplexes (372 ± 18 nm),^[^ ^5^ ^]^ attributed to the polymer lipid hybrid composition (Figure 1b; Tables S1 and S2, Supporting Information). Similar negative Z‐potential values were measured for PLGA NPs and hybrid NPs (−28 ± 2 and −20 ± 2 mV, respectively), while DE‐DOPE/miRNA lipoplexes showed a positive Z‐potential (40 ± 12 mV; Figure 1b; Tables S1 and S2, Supporting Information).^[^ ^5^ ^]^ Hybrid NPs showed a monodisperse size distribution (Tables S1 and S2, Supporting Information) with a PDI of 0.27 ± 0.05, higher than that of PLGA NPs (0.11 ± 0.02).^[^ ^5^ ^]^ Compared to hybrid NPs, commercial RNAiMAX NPs showed a higher hydrodynamic diameter of 806 ± 37 nm (p‐value < 0.0001), a higher PDI of 0.34 ± 0.02, and a positive Z‐potential of 56 ± 2 mV (Figure 1b; Table S2, Supporting Information). LE of negmiR‐loaded hybrid NPs was 99% ± 1, significantly higher compared to RNAiMAX NPs (57% ± 3, p < 0.0001) (Figure 1c). Control PLGA NPs did not load negmiR (data not reported in Figure 1c).

NegmiR‐ and miRcombo‐loaded hybrid NPs showed similar controlled and sustained release kinetics in phosphate‐buffered saline solution (PBS) at 37 °C under dynamic conditions, reaching a miRNA release of ≈50% after 48 h and of 100% after 9 days (Figure 1d). In contrast, negmiR‐loaded RNAiMAX NPs showed a faster release kinetics in the same conditions, reaching 85% miRNA release at 48 h and complete miRNA release at 72 h.

Moreover, morphological analysis of hybrid NPs, performed by Cryogenic Transmission Electron Microscopy (Cryo‐TEM), showed that hybrid NPs had a spherical shape and an average size in agreement with data from Dynamic Light Scattering (DLS) analysis, did not form aggregates and displayed a more electron‐dense core and a less dense outer shell, suggesting the presence of a core‐shell structure (Figure 1e). Nanoparticle Tracking Analysis (NTA) confirmed hybrid NP size (Figure 1f) and allowed the measurement of hybrid NP suspension concentration, resulting in equal to 2.14 × 10^11^ particles/mL. Production yield of hybrid NPs, calculated as the weight of the produced NPs referred to over the weight of starting materials, showed a high value of 97 ± 1%.

**Figure 2:**
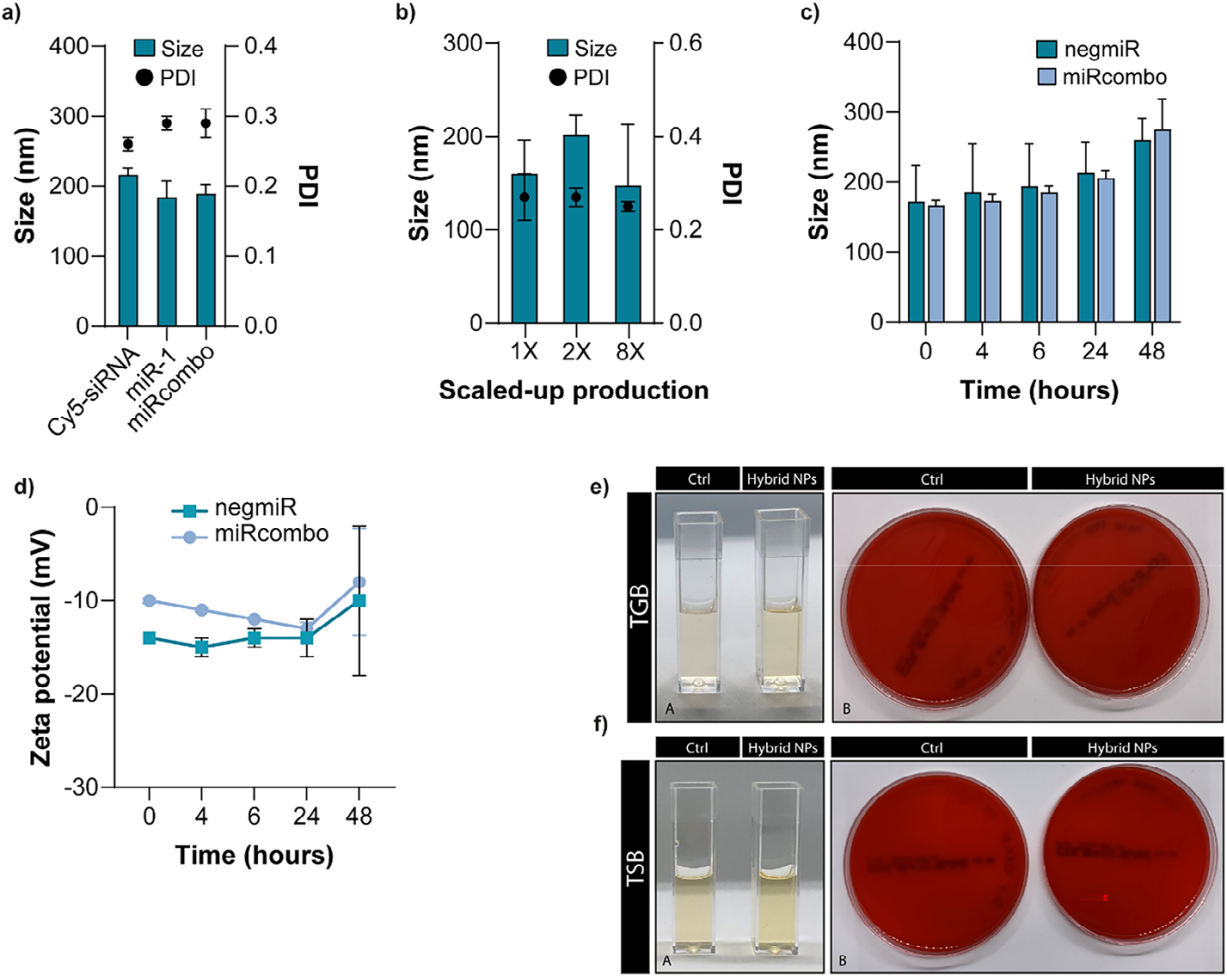
Physicochemical properties of hybrid NPs incubated in different media, scalability of the production process, and sterility of hybrid NPs. a) Hydrodynamic diameter and PDI of hybrid NPs loaded with different oligonucleotides (Cy5‐siRNA, miR‐1, and miRcombo) measured by DLS. b) Hydrodynamic diameter and PDI of hybrid NPs prepared using 1, 2, and 8X volumes and measured by DLS. c,d) Stability study at physiological temperature in DMEM+FBS by measuring: hydrodynamic size (c) and Z‐potential (d) of hybrid NPs as a function of their incubation time (0, 4, 6, 24, and 48 h). e,f) Analysis of microbial contamination of freshly prepared negmiR‐loaded hybrid NPs kept in TBG at 37 °C (e) and TSB at 25 °C (f) for 14 days. In both figures f,g) the images on the left show turbidity assessment by visual inspection (i.e., cuvettes with clear broth indicating the absence of microbial contamination), while the images on the right show the absence of CFU on agar solid plates. Control (Ctrl) samples are TGB and TSB with no hybrid NPs, respectively. Data are expressed as mean ± SD. Statistical analysis was performed by a two‐sided *t*‐test.

The same formulation method was used to prepare hybrid NPs loaded with Cy5‐siRNA, miR‐1, and miRcombo. Resulting hybrid NPs showed similar hydrodynamic diameter, PDI, and Z‐potential compared to previously characterized negmiR‐loaded hybrid NPs (**Figure** **2**a; Table S3, Supporting Information).

The scalability of hybrid NPs production process using up to 8‐fold (8X) higher volumes was then demonstrated based on similar DLS properties and LE values (Figure 2b; Table S4, Supporting Information).

### 2.2 Physicochemical Characterization of Hybrid NPs Incubated in Different Media at Physiological Temperature

The stability of negmiR‐ and miRcombo‐loaded hybrid NPs was evaluated overtime for up to 48 h at 37 °C, using different media (i.e., Milli‐Q water and PBS at pH 7.4 Figure S2 and Table S5 (Supporting Information) and Dulbecco Modified Eagle Medium supplemented with 10% Fetal Bovine Serum, DMEM+FBS Figure 2c,d). Hybrid NPs incubated in Milli‐Q water showed a stable hydrodynamic diameter, Z‐potential, and PDI. Similar behavior was observed for hybrid NPs incubated in PBS, with a slight non‐significant increase in hydrodynamic diameter, PDI, and Z‐potential after 48 h (Figure S2 and Table S5, Supporting Information). Incubation in DMEM+FBS did not cause significant changes in the size, hydrodynamic diameter, and PDI of negmiR‐ and miRcombo‐loaded hybrid NPs up to 24 h (Figure 2c,d; Table S5, Supporting Information). Notably, the Z‐potential of hybrid NPs in DMEM+FBS was slightly higher than in Milli‐Q water (i.e., ≈−14 vs −24 mV, respectively), possible due to the protein corona formation. A slight but non‐significant increase in both the average hydrodynamic size and Z‐potential of negmiR‐ and miRcombo‐loaded hybrid NPs was observed after 48 h incubation in DMEM+FBS (Figure 2c,d; Table S5, Supporting Information), attributed to the absorption of serum proteins. PDI values did not change significantly during incubation in DMEM+FBS up to 48 h (0.25–0.26) suggesting a good colloidal stability for negmiR‐ and miRcombo‐loaded hybrid NPs, which is important for their use as in vitro transfection reagents.

Next, we investigated whether the hybrid NPs preparation method could preserve NPs sterility. The absence of microbial contamination in negmiR‐loaded hybrid NPs was investigated through the direct inoculation method by culturing samples in Tryptic‐Soya Broth (TSB, for aerobic bacteria) at 25 °C and Thioglycolate Broth (TGB, for anaerobic bacteria) at 37 °C for 14 days. After 1, 7, and 14 days of incubation, no bacterial growth was detected in both broths by turbidity analysis, through visual inspection and the evaluation of lack of absorbance at 457, 490, and 600 nm (data non‐reported), and by Colony Forming Unit (CFU) analysis (Figure 2e,f), suggesting the sterility of hybrid NPs.

### 2.3 In Vitro Cell Characterization of miRNA‐Loaded Hybrid NPs: In Vitro Uptake, Transfection Efficiency and Proof of Concept Direct Reprogramming of AHCFs

**Figure 3:**
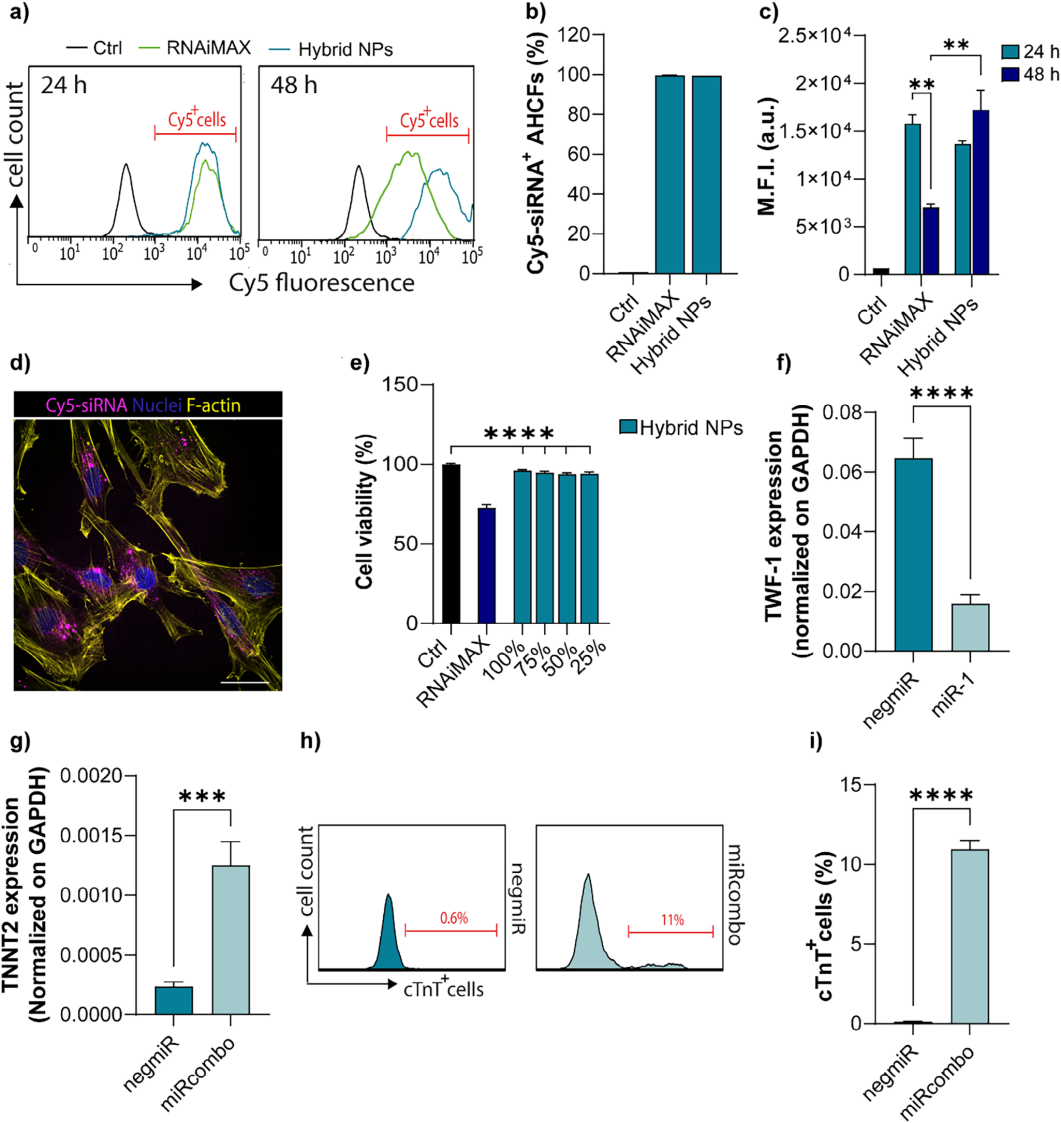
Hybrid NPs showed efficient uptake, miRNA release, and gene expression regulation in AHCFs. a) Representative flow cytometry analysis showing cellular uptake of hybrid NPs and RNAiMAX loaded with Cy5‐siRNA in AHCFs after 24 and 48 h of treatment. b) Bar graphs showing the percentage of Cy5‐siRNA positive cells (Cy5‐siRNA^+^ cells) after 24 h treatment with hybrid NPs and RNAiMAX. c) M.F.I. after 24 h and 48 h of treatment with hybrid NPs and RNAiMAX. Untreated cells were used as control for all experiments. d) Representative fluorescence microscopy images showing Cy5‐siRNA (magenta) uptake by AHCFs, mediated by hybrid NPs after 24 h treatment. Nuclei were counterstained with DAPI (blue) and F‐actin with Phalloidin (yellow). Scale bar = 50 µm. e) Viability of AHCFs treated with negmiR‐loaded hybrid NPs and RNAiMAX NPs at 24 h. Hybrid NPs were tested at four concentrations: 100, 75, 50, and 25 (%) (Table 1). Cell viability was analyzed by resazurin assay. The viability of transfected AHCFs was normalized to that of non‐transfected AHCFs. f) Gene expression of TWF‐1 mRNA target in AHCFs treated with negmiR or miR‐1‐loaded hybrid NPs at 48 h, analyzed by ddPCR. g) Gene expression of TNNT2 mRNA in AHCFs treated with negmiR or miRcombo‐loaded hybrid NPs after 15 days of culture post‐treatment, analyzed by ddPCR. h,i) Representative flow plots (h) and percentage (i) of cTnT+ cells for AHCFs treated with negmiR or miRcombo using hybrid NPs after 15 days of culture. Reported data are average values ± SEM of three independent experiments. Statistical differences between the groups were determined by two‐sided *t*‐tests.

In vitro cell tests were performed using AHCFs. AHCFs were incubated with Cy5‐siRNA loaded hybrid NPs and RNAiMAX NPs for 24 h, to evaluate cell uptake. Flow cytometry analysis, performed at 24 and 48 h revealed that 100% AHCFs were positive for Cy5‐siRNA (% of Cy5‐siRNA^+^ cells) (**Figure** **3**a,b). Mean fluorescent intensity (M.F.I.) of Cy5‐siRNA within cells was similar for AHCFs treated with both hybrid or RNAiMAX NPs after 24 h, while significantly lower M.F.I was recorded for AHCFs treated with RNAiMAX NPs after 48 h (*p*‐value = 0.003, Figure 3c). Efficient uptake of Cy5‐siRNA loaded hybrid NPs was also confirmed by confocal microscopy analysis, showing fluorescent Cy5‐siRNA signal inside the cell cytoplasm after 24 h (Figure 3d). The ability of hybrid NPs to ensure Cy5‐siRNA endosome/lysosome escape was also determined. AHCFs were incubated with Cy5‐siRNA loaded in hybrid NPs and RNAiMAX NPs for 24 h. Confocal images revealed that disperse miRNA fluorescence signal in the cytoplasm at 24 h was observed, with little colocalization of endosomes/lysosomes (Figure S3.1, Supporting Information). Pearson's correlation coefficient (PCC) was performed to assess the degree of colocalization between lysosomes and the Cy5‐siRNA loaded NPs. The results indicate low colocalization for both RNAiMAX NPs (0.35 ± 0.13) and hybrid NPs (0.39 ± 0.03) at 24 h post‐transfection, suggesting that both formulations exhibit efficient lysosomal escape (Figure S3.1, Supporting Information). No significant decrease in cell viability was observed after 24 h treatment of AHCFs with different concentrations of hybrid NPs (**Table** **1**), while cells transfected with RNAiMAX NPs prepared following the manufacturer's instructions, showed a decreased viability of 70% (*p*‐value < 0.0001, Figure 3e). Results indicate the superior biocompatibility and cell uptake ability of miRNA‐loaded hybrid NPs, over the commercial transfectant RNAiMAX. To study if hybrid NPs may induce the expression of pro‐inflammatory cytokines, we analyzed the expression of Interleukin‐6 (IL‐6) at 24 h post‐transfection. Despite DE‐DOPE/negmiR transfection significantly increased IL‐6 expression compared to control cells, the PLGA shell present in hybrid NPs was able to restore IL‐6 expression levels as for control cells and cell transfected with RNAiMAX (Figure S3.2, Supporting Information). The transfection efficiency of hybrid NPs loaded with miR‐1 and negmiR was studied by measuring the expression of Twinfilin‐1 (TWF‐1) mRNA (which represent a mRNA target for miR‐1) in AHCFs by droplet digital PCR (ddPCR).^[^ ^5^, ^6^, ^7^, ^8^, ^9^, ^10^ ^]^ AHCFs treated with hybrid NPs loaded with miR‐1 efficiently induced TWF‐1 downregulation (p = 0.0006), showing nearly 25% of mRNA expression compared to negmiR loaded hybrid NPs (Figure 3f). AHCFs were also treated with hybrid NPs loaded with miRcombo (a combination of four miRNAs), able to directly reprogram AHCFs into induced cardiomyocytes (iCMs).^[^ ^2^, ^3^, ^5^, ^7^, ^10^ ^]^ High expression of TNNT2 mRNA was measured for AHCFs treated with miRcombo compared to negmiR loaded hybrid NPs (*p*‐value = 0.0001, Figure 3g). Flow cytometry analysis was performed to measure cTnT+ cells as an index of direct reprogramming efficiency of AHCFs into iCMs. A percentage of ≈11% cTnT+ cells was measured for cells treated with miRcombo‐loaded hybrid NPs, while cells did not express cTnT when treated with hybrid NPs loaded with negmiR (*p*‐value < 0.0001, Figure 3h,i).

**Table 1:** miRNA dose administered through hybrid NPs at different dilutions for in vitro cytocompatibility and cytotoxicity studies.

Hybrid NPs Concentration	miRNA Concentration [nm] per well	miRNA dose (pmol) per well volume (100 µL)
100%	25	5
75%	18.75	3.75
50%	12.50	2.50
25%	6.25	1.25

### 2.4 Long‐Term Storage of Hybrid NPs Without Alterations in Physicochemical, Functional Properties and Sterility

**Figure 4:**
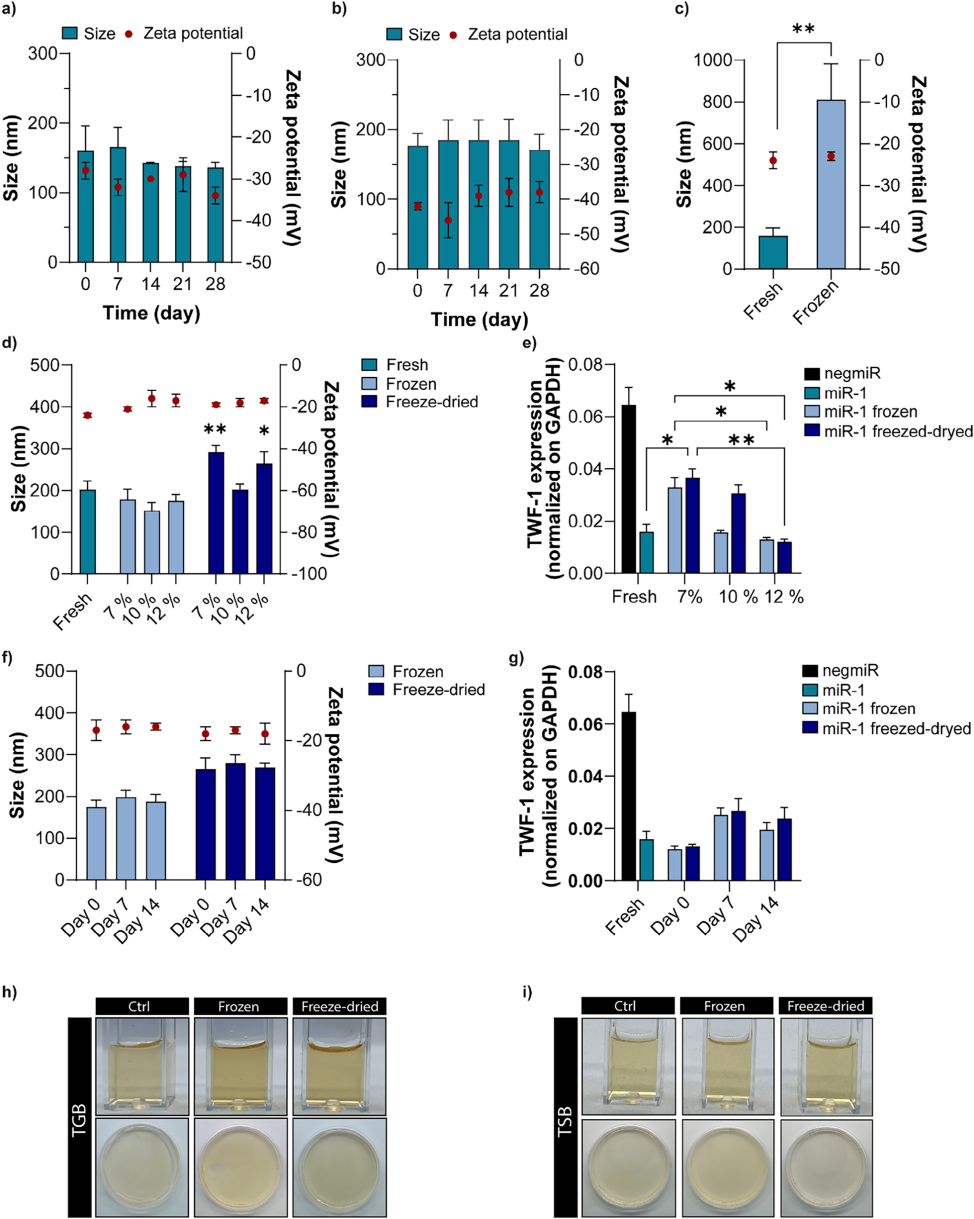
Stability and microbial contamination analysis of hybrid NPs under storage conditions. a,b) Hydrodynamic diameter and Z‐potential of hybrid NPs stored in RNase‐free water suspension at 4 °C encapsulating (a) negmiR and (b) miRcombo evaluated over time (0, 7, 14, 21, and 28 days). c) Hydrodynamic diameter and Z‐potential of frozen hybrid NPs loaded with negmiR compared to fresh hybrid NPs. Statistical analysis was performed by two‐sided *t*‐test. d) Hydrodynamic diameter and Z‐potential of frozen and freeze‐dried hybrid NPs with different concentrations of trehalose (7, 10, and 12% w/v) compared to fresh hybrid NPs. e) Gene expression of TWF‐1 mRNA target (normalized on GAPDH) in AHCFs transfected with negmiR or miR‐1 using fresh, frozen, and freeze‐dried hybrid NPs with different concentrations of trehalose (7, 10, and 12% w/v). Gene expression was analyzed at 48 h post‐transfection by ddPCR. f) Hydrodynamic diameter and Z‐potential of frozen and freeze‐dried hybrid NPs with 12% w/v of trehalose evaluated over time (0, 7, and 14 days). g) Gene expression of TWF‐1 mRNA target in AHCFs transfected with negmiR or miR‐1 using frozen and freeze‐dried hybrid NPs with 12% w/v of trehalose after 0, 7, and 14 days of storage compared to fresh hybrid NPs. Gene expression was analyzed at 48 h post‐transfection by ddPCR. h,i) Analysis of microbial contamination of frozen and freeze‐dried negmiR hybrid NPs stored for 14 days cultured in TBG at 37 °C (h) and TSB at 25 °C (i) for 14 days. In both figures, the images at the top represent the turbidity assessment (i.e., cuvettes with clear broth indicating the absence of microbial contamination), while the images at the bottom show the absence of CFU on agar solid plates. Ctrl samples are TGB and TSB with no hybrid NPs, respectively. For physicochemical hybrid NPs characterizations, data are expressed as mean ± SD of three independent experiments. Statistical analysis was performed by 1‐way ANOVA, unless otherwise specified in the text. For in vitro cells study, data are expressed as mean ± SEM of three independent experiments. Statistical analysis was performed by two‐sided t‐test.

The long‐term physical stability of hybrid NPs loaded with negmiR and miRcombo was studied by measuring the hydrodynamic diameter, PDI, and Z‐potential after their incubation in RNase‐free water at 4 °C for 0, 7, 14, 21, and 28 days (**Figure** **4**a,b; Table S6, Supporting Information). The hydrodynamic diameter was stable over time for both negmiR (136–166 nm) and miRcombo (171–185 nm) loaded hybrid NPs. The negative Z‐potential and PDI values (Table S6, Supporting Information) did not vary over 4 weeks for both negmiR‐ and miRcombo‐loaded hybrid NPs. Then, the transfection efficiency of miR‐1 loaded hybrid NPs after their storage at 4 °C at different time points (0 h, 48 h, 7, and 14 days) was evaluated in AHCFs by analyzing TWF‐1 expression by ddPCR. TWF‐1 downregulation was measured in all tested conditions (Figure S3.3, Supporting Information) compared to negmiR‐loaded hybrid NPs, confirming the ability of hybrid NPs to preserve miRNA biological activity up to 14 days incubation in RNase‐free water at 4° C. However, freshly prepared hybrid NPs showed a higher TWF‐1 mRNA downregulation compared to hybrid NPs stored for 7 and 14 days at 4 °C (*p* value = 0.0002 and 0.0015, respectively). Therefore, keeping hybrid NPs in suspension at 4 °C was found not to be a suitable method for their long‐term storage. For this reason, freezing and freeze‐drying methods were studied as alternative long term methods with the aim to preserve the physicochemical characteristics and miRNA activity of hybrid NPs.^[^ ^56^ ^]^ Hybrid NPs, initially frozen at −80 °C, then thawed and resuspended, showed a significant increase in size, reaching 811 nm (Figure 4c), with no changes in Z‐potential (Figure 4c) and PDI (Table S7, Supporting Information). To improve stability and reduce the increment in size during the freezing process, trehalose was added as cryoprotectant.^[^ ^57^, ^58^, ^59^, ^60^ ^]^ As the drying process induced an additional destabilization, freeze‐drying method was first evaluated as a long term storage method by optimizing the trehalose concentration and then, freezing was assessed as other preservation approach.^[^ ^56^, ^61^ ^]^

NegmiR‐loaded hybrid NPs freeze‐dried in the presence of different trehalose concentrations (2.5, 5, 7, 10, 12, 13, 15, and 18% w/v) were re‐dispersed in RNase‐free water and characterized for their hydrodynamic size and Z‐potential (Figure S4, Supporting Information). The hydrodynamic diameter of rehydrated hybrid NPs after freeze‐drying with 0% and 10% w/v trehalose, compared to freshly prepared NPs, was found to be dependent on the amount of cryoprotectant. Indeed, a limited (i.e., up to 7% w/v) or excessive (i.e., > 12% w/v) trehalose concentration could not preserve the hydrodynamic size of rehydrated hybrid NPs (Figure S4, Supporting Information). An increase in cryoprotectant amount also increased the Z‐potential values, due to the positive charge of trehalose covering the hybrid NP surface (Figure S4, Supporting Information). No variations in PDI values were observed as a function of cryoprotectant dose (Table S8, Supporting Information). Based on these data, a trehalose concentration from 7 to 12% w/v was selected for freezing method and in vitro studies.

Hybrid NPs with 7, 10, and 12% w/v trehalose preserved their physicochemical properties after the freezing and freeze‐drying process followed by reconstitution of NPs suspensions compared to freshly prepared NPs (Figure 4d; Tables S8 and S9, Supporting Information). AHCFs showed high cytocompatibility after treatment with hybrid NPs, previously frozen or freeze‐dried for 24 h in the presence of 7, 10, and 12% w/v trehalose (Figure S5a, Supporting Information). On the other hand, trehalose concentration and the adoption of frozen or freeze‐drying treatment were found to affect miR‐1 activity and TWF‐1 downregulation. Specifically, AHCFs incubated for 24 h with miR‐1‐loaded hybrid NPs, reconstituted after freezing or freeze‐drying with 12% w/v of trehalose, displayed TWF‐1 downregulation levels similar to the one achieved by treatment with fresh hybrid NPs (Figure 4e). Treatment of AHCFs with hybrid NPs, reconstituted after freezing or freeze‐drying with 7 and 10% w/v trehalose, decreased TWF‐1 mRNA expression compared to negmiR‐loaded hybrid NPs (*p* value = 0.002 and 0.0001, respectively). However, TWF‐1 mRNA expression levels in AHCFs were significantly higher upon treatment with NPs with 7% w/v trehalose (*p* value = 0.0194) and slightly higher (although not significantly) upon treatment with NPs with 10% w/v trehalose, compared to freshly prepared NPs and previously frozen or freeze‐dried hybrid NPs with 12% w/v trehalose. Therefore, 12% w/v trehalose was selected as the optimal cryoprotectant concentration for maintaining the physicochemical properties and miRNA activity of hybrid NPs during freezing and freeze‐drying.

To study if long‐term storage may affect NPs properties, frozen and freeze‐dried hybrid NPs with 12% w/v of trehalose were stored respectively at −80 and −20 °C for 7 and 14 days. After suspension reconstitution following storage, hybrid NPs exhibited unchanged hydrodynamic size, Z‐potential, and PDI (Figure 4f; Table S10, Supporting Information), suggesting the maintenance of their physicochemical properties under storage. Furthermore, AHCFs treated with frozen or freeze‐dried hybrid NPs, reconstituted after storage for 7 and 14 days, displayed 100% cell viability (Figure S5b, Supporting Information). In addition, AHCFs treated with hybrid NPs loaded with miR‐1, reconstituted after storage for 0, 7, and 14 days, displayed lower TWF‐1 mRNA expression (Figure 4g) compared to freshly prepared hybrid NPs loaded with negmiR (*p* value = 0.0001) and similar TWF‐1 mRNA expression compared to freshly prepared miR‐1 loaded hybrid NPs.

Finally, the sterility of negmiR‐loaded hybrid NPs was also investigated upon storage for 0, 7, and 14 days, in both frozen and freeze‐dried form, by evaluating the presence of microbial contamination. Bacterial growth was studied in TSB at 25 °C and in TGB at 37 °C for 14 days by turbidity observation and CFU analyses, as previously performed for freshly prepared NPs without cryoprotectant (Figure 2f,g). After 0, 7, and 14 days storage, both frozen and freeze‐dried hybrid NPs with 12% w/v trehalose showed no sign of bacterial contamination upon incubation in TSB and TGB, as indicated by absence of CFU on agar plates and turbidity measurements through visual inspection as well as lack of absorbance bands at 457, 490, and 600 nm (data non reported), suggesting the maintenance of their sterility under storage (Figure 4h,i).

## 3 Discussion

In this work, hybrid NPs based on lipids (DE‐DOPE) and a synthetic polymer (PLGA) were designed for miRNA delivery (Figure 1a), as novel transfection vectors for research use, with improved properties compared to lipidic transfectants, such as commercial RNAiMAX NPs^[^ ^16^, ^32^ ^]^ and DharmaFECT1^[^ ^2^, ^6^, ^34^, ^35^ ^]^ and previously optimized DE‐DOPE lipoplexes.^[^ ^5^ ^]^ Indeed, in a previous work, we formulated DE‐DOPE/miRNA lipoplexes (with DE:DOPE 50:50 w:w ratio; amino to phosphate group ratio – N/P – of 3) for miRNA delivery, showing an hydrodynamic diameter of 372 ± 18 nm, a positive Z‐potential of 40 ± 12 mV, a PDI of 0.29 ± 0.08 and LE of 99%.^[^ ^5^ ^]^ Although highly biocompatible and efficient in cell transfection, DE‐DOPE/miRNA lipoplexes suffer from poor stability in physiological media, completely releasing their cargo within 24 h incubation in PBS.^[^ ^5^ ^]^ Due to their limited colloidal stability, DE‐DOPE/miRNA lipoplexes cannot be stored in suspension after preparation and require an immediate use similar to the main lipid‐based commercial transfection agents. Furthermore, they cannot be loaded into hydrogels for a controlled and sustained release, due to their rapid collapse in contact with hydrogel molecules (data not reported). To address such limitations, in this work hybrid NPs with improved stability were designed by a simple nanoprecipitation method (Figure 1a). The cationic nature of DE‐DOPE lipoplexes (Z‐potential of 40 ± 12 mV) and their amphiphilic properties allowed efficient interaction with PLGA.^[^ ^5^, ^10^, ^50^, ^62^ ^]^ Indeed, PLGA (Resomer RG 752 H), a biocompatible and biodegradable co‐polymer approved by US FDA (Food & Drugs Administration), is composed by both hydrophilic (glycolic acid, 25%) and hydrophobic (lactic acid, 75%) monomer units and, being carboxyl acid‐terminated, is negatively charged. Such characteristics favor PLGA electrostatic interaction with DE‐DOPE lipoplexes. A PLGA content of 95% w/w was selected for hybrid NPs preparation, in order to get PLGA surface distribution, as suggested by negative Z‐potential (−28 ± 2 mV) compared to PLGA NPs (non‐encapsulating miRNA) (Table S2, Supporting Information; Figure 1b). On the contrary, hybrid NPs with a PLGA content of 70, 75, and 78% showed a larger hydrodynamic diameter of ≈400 nm and neutral Z‐potential, suggesting an incomplete PLGA covering (Figure S1 and Table S1, Supporting Information). Cryo‐TEM analysis of hybrid NPs with selected composition (Figure 1e) showed their spherical shape, the lack of aggregates, and the presence of a more electron‐dense core compared to the outer shell which might support the hypothesis of a lipoplex core PLGA shell structure. The concentration of produced hybrid NPs measured by NTA was 2.14 × 10^11^ particles/mL. Previously, hybrid NPs with a lipoplex or polyplex core and a PLGA shell have been only produced by complex and time‐consuming double emulsion‐solvent evaporation methods.^[^ ^44^, ^48^, ^49^, ^50^, ^53^, ^55^, ^63^ ^]^ In this work, nanoprecipitation was proposed as a simple, scalable, user‐friendly, and more environmentally friendly approach, avoiding the use of potentially cytotoxic surfactants.^[^ ^45^ ^]^ Nanoprecipitation is a simple process, compatible for the preparation of transfection vectors for research use. An in‐depth physicochemical characterization of hybrid NPs was performed versus Lipofectamine RNAiMAX, a widely used lipid commercial transfection agent. Hybrid NPs showed a significantly lower size than RNAiMAX NPs (160 ± 36 nm vs. 806 ± 37 nm, respectively; Figure 1b; Tables S2 and S3, Supporting Information) and a negative versus positive Z‐potential (−28 ± 2 mV vs 56 ± 2 mV, respectively; Figure 1b; Tables S2 and S3, Supporting Information). Hybrid NPs showed a significantly higher LE than RNAiMAX NPs (99% ± 1 vs 57% ± 3, Figure 1c), attributed to the complete incorporation of DE‐DOPE lipoplexes showing efficient miRNA loading ability.^[^ ^5^ ^]^ LE of RNAiMAX NPs was lower and close to that of commercial DharmaFECT NPs (65% ± 1) reported in our previous study.^[^ ^5^ ^]^ Compared to commercial transfection agents, higher miRNA loading in hybrid NPs may reduce the nanocarrier dosage to get a certain biological effect. Complete miRNA loading also avoids any waste of miRNA molecules. Additionally, the production yield of hybrid NPs was high (97% ± 1) minimizing miRNA, polymer, and DE‐DOPE waste, in agreement with environmental sustainability principles. Hybrid NPs allowed the loading of single miRNAs (such as miR‐1 and negmiR), fluorescently labeled oligonucleotides (Cy5‐siRNA), and miRNA combinations (miRcombo) preserving their physicochemical properties (Figure 2a; Table S3, Supporting Information). The method for hybrid NPs preparation was scalable allowing up to 8X production for intensive laboratory experiments, without any alteration in hybrid NPs physicochemical properties and LE (Figure 2b; Table S4, Supporting Information). Thanks to their composition and relative distribution of components, hybrid NPs showed high stability in different media, such as Milli‐Q water, PBS, and DMEM+FBS, at 37 °C over 48 h (Figure 2c,d; Figure S2 and Table S5, Supporting Information). Only a slight but non‐significant increase in the average hydrodynamic diameter and Z‐potential of negmiR‐ and miRcombo‐loaded hybrid NPs was measured upon incubation in PBS (Figure S2 and Table S5, Supporting Information) and DMEM+FBS (Figure 2c,d; Table S5, Supporting Information) after 48 h, attributed to the interaction with ionic species and serum proteins, respectively. PDI values (0.25–0.26) did not change during incubation in DMEM+FBS up to 48 h, suggesting a good colloidal stability for hybrid NPs, important for their use as in vitro transfection vectors. Conversely, previous experiments have shown that DE‐DOPE/miRNA lipoplexes and DharmaFECT NPs maintain stable size and Z‐potential only up to 6 and 24 h incubation in Milli‐Q water at 37 °C,^[^ ^5^ ^]^ disassembling in 6–24 and 24–48 h incubation in Milli‐Q water, respectively. The superior stability of hybrid NPs in simple (Milli‐Q water) and complex media (PBS and DMEM+FBS) compared to lipoplexes is expected to limit miRNA release in the time before NPs are internalized by cells and to result in a more efficient intracellular miRNA release. This hypothesis was supported by in vitro miRNA release kinetics experiments. Indeed in vitro miRNA delivery from hybrid NPs was controlled and sustained up to 9 days of incubation in PBS under dynamic conditions (Figure 1d). On the opposite, RNAiMAX NPs were characterized by a faster miRNA release kinetics under dynamic conditions, reaching 85% miRNA release after 48 h and complete miRNA release at 72 h.

Optimal storage conditions of hybrid NPs were investigated to support their use for preclinical research studies. Due to their poor colloidal stability, lipid NPs cannot be stored in water suspension at low temperatures as they tend to rapidly collapse or aggregate.^[^ ^56^, ^64^ ^]^ In contrast, hybrid NPs could be stored in water suspension at 4 °C for up to 4 weeks preserving their physicochemical properties (Figure 4a,b). However, their transfection efficiency was retained only for short incubation time, up to 48 h (Figure S3.3, Supporting Information). These findings demonstrated that hybrid NPs can be stored in water‐based suspension at 4 °C at short‐term (< 48 h) after their preparation (Figure S3.3, Supporting Information). For long‐term storage of hybrid NPs, freezing and freeze‐drying methods were investigated as better options.^[^ ^56^, ^61^, ^64^ ^]^ To avoid possible NP agglomeration phenomena during freezing (Figure 4c) and freeze‐drying, cryo‐ and lyoprotectants have been exploited.^[^ ^64^, ^65^, ^66^ ^]^ Several studies have demonstrated that trehalose is an optimal cryoprotectant, able to efficiently stabilize different types of nanoparticles, including PLGA‐based ones.^[^ ^57^, ^58^, ^59^, ^60^ ^]^ Cryoprotectant concentration is a crucial parameter for NPs stabilization: a minimum concentration is needed to stabilize the physicochemical properties of NPs,^[^ ^60^, ^67^, ^68^ ^]^ while concentrations exciding a threshold may destabilize NPs.^[^ ^58^, ^60^ ^]^ In this work, a trehalose concentration ≤ 7% w/v was not able to stabilize freeze‐dried hybrid NPs (Figure S4, Supporting Information; Figure 4d). Conversely, at a trehalose concentration > 12% w/v, freeze‐dried hybrid NPs showed a statistically significant increase in size (Figure S4, Supporting Information; Figure 4d). The presence of trehalose did not alter the cytocompatibility of hybrid NPs (Figure S5, Supporting Information), while it affected in vitro transfection efficiency depending on the applied freezing or freeze‐drying methods (Figure 4e). As the drying process induced an additional destabilization,^[^ ^56^, ^61^ ^]^ optimal minimal cryoprotectant concentration was higher for freeze‐dried (12% w/v) with respect to frozen hybrid NPs (10% w/v) (Figure 4f). A trehalose concentration of 12% w/v was thus selected for both treatments and storage experiments were performed. The physicochemical properties (Figure 4f), biocompatibility (Figure S5, Supporting Information), transfection efficiency for AHCFs (Figure 4g), and sterility (Figure 4h,i) were preserved for reconstituted hybrid NPs after storage for up to 14 days. Although freezing and freeze‐drying methods were both suitable for long‐term storage of hybrid NPs, freeze‐drying is more advantageous as it reduces sample volume and weight and allows an easy modulation of reconstituted NP suspension concentration after storage.^[^ ^64^, ^67^, ^69^ ^]^

Finally, hybrid NPs were thoroughly studied for use as in vitro transfection vectors for miRNA delivery to AHCFs compared to RNAiMAX NPs. Transfection vectors for AHCFs are demanded in several preclinical studies, such as miRNA‐mediated modulation of fibrotic cell phenotype or cell reprogramming, e.g., the direct reprogramming of fibroblasts into CMs. Attributed to their negative Z‐potential, hybrid NPs showed superior biocompatibility compared to positively charged RNAiMAX NPs (Figure 3e) and previously reported DharmaFECT NPs.^[^ ^5^ ^]^ After 24 h treatment of AHCFs with hybrid NPs or RNAiMAX NPs loaded with Cy5‐siRNA, 100% cells internalized the fluorescent oligonucleotide in the cell cytoplasm (Figure 3a–d). However, fluorescence intensity of cells treated with hybrid NPs further slightly increased at 48 h, while it significantly decreased upon RNAiMAX NPs treatment (Figure 3c), suggesting a sustained and controlled versus a burst intracellular miRNA delivery, respectively. Transfection efficiency of hybrid NPs for AHCFs was demonstrated by delivering miR‐1, through the decreased expression of target TWF‐1 mRNA (Figure 3f) in AHCFs. Hybrid NPs efficacy was further assessed in a miRcombo model of fibroblast‐to‐cardiomyocyte reprogramming. Proof of concept tests of direct reprogramming of AHCFs using miRcombo‐loaded versus negmiR‐loaded hybrid NPs showed that miRcombo treatment induced a significantly higher expression of cTnT at gene (*p* value = 0.0001, Figure 3g) and protein level (Figure 3h,i), after 15 days culture, resulting in 11% reprogramming efficiency. As a future perspective, achieving full functional maturation of iCMs remains a significant challenge.^[^ ^15^ ^]^ Multifactorial approaches, such as scaffold engineering, electrical stimulation, and anisotropic mechanical cues, will be necessary to enhance sarcomere organization and contractility.^[^ ^70^ ^]^ These strategies may help overcome the barriers that limit complete cardiac reprogramming.

Overall results from this work demonstrated the potentialities of hybrid NPs for further exploitation at an industrial scale as novel in vitro transfection vectors for research use, with proof of concept results for efficient miRNA transfection of AHCFs. As a future perspective, hybrid NPs may represent a promising platform for in vivo therapeutic applications, by improving their stability, thanks to the addition of anti‐fouling polymers such as PEG,^[^ ^71^ ^]^ and cell‐targeting capability by performing surface functionalization with targeting molecules, such as using organ and cell‐specific antibody or peptide.^[^ ^72^ ^]^ The goal will be to develop effective and safe targeted delivery systems for advanced therapies, particularly in the cardiac field.

## 4 Conclusion

Hybrid NPs, prepared by a novel simple, user friendly, high‐yield, and scalable nanoprecipitation method, were herein demonstrated to be optimal transfection vectors for in vitro miRNA delivery, combining the complete miRNA LE of DE‐DOPE lipoplexes with the colloidal stability and gradual and sustained miRNA release ability of PLGA NPs. Hybrid NPs overcame the main limitations of cationic lipid NPs, showing controlled and sustained in vitro miRNA delivery and long‐term storage ability upon freeze‐drying, in the presence of trehalose, followed by maintenance at −20 °C, recovering their physicochemical properties and transfection ability after rehydration. AHCFs treated with hybrid NPs, loaded with miRNAs or model fluorescent siRNAs, showed higher cell viability and efficient NPs internalization with high intracellular miRNA level at 48 h, compared to commercial lipidic transfectant RNAiMAX. Proof of concept experiments showed that hybrid NPs were able to efficiently transfect AHCFs with both single miRNAs and miRNA combinations, such as miRcombo, inducing early in vitro direct reprogramming into iCMs. The versatility of hybrid NPs could allow their exploitation for the transfection of different cells with other miRNAs or siRNAs, in order to study oligonucleotide therapeutic effect at the preclinical level. Scalability, high‐yield, environmentally friendliness and simplicity of the preparation method, colloidal stability of hybrid NP suspensions, and their storage ability are promising properties for the application of hybrid NPs as new in vitro transfection vectors to be exploited at an industrial scale.

## 5 Experimental Section

### Preparation of Hybrid and Control NPs

DE/DOPE liposomes (([2‐(2,3‐didodecyloxypropyl)‐hydroxyethyl] ammonium bromide, DE^[^ ^73^ ^]^ and L‐alpha‐dioleoyl phosphatidylethanolamine, DOPE, Merck), in a 1:1 w/w ratio were prepared according to the thin lipid film‐hydration method.^[^ ^5^, ^10^ ^]^ Briefly, the cationic lipid DE and the helper lipid DOPE were dissolved separately in chloroform and dried under reduced pressure in a rotary evaporator. The dried lipid film was dissolved in RNase‐free water (Fisher Bioreagents) to a final concentration of 1 mg mL^−1^ of the liposomal suspension. DE‐DOPE/miRNA lipoplexes were prepared at amino to phosphate group molar ratio (N/P) equal to 3, according to a previously optimized protocol.^[^ ^5^, ^10^ ^]^ Specifically, 10 µL miRNA solution (5 µm) and 6 µL DE‐DOPE suspension were incubated for 20 min at room temperature, vortexed, and then incubated at room temperature for additional 20 min. Then, lipoplexes were diluted in RNase‐free water to a final volume of 1 mL. DE‐DOPE/miRNA lipoplexes were loaded with different oligonucleotides, such as negmiR (mirVana miRNA Mimic, Negative Control #1, Life Technologies), miR‐1, miRcombo (miR‐1‐3p, miR‐133a‐3p, miR‐208a‐3p, and miR‐499a‐5p, mirVana miRNA mimic, Life Technologies) or Cy5‐siRNA (MISSION siRNA Fluorescent Universal Negative Control #1, Cyanine 5, Sigma Aldrich).

Hybrid NPs loaded with oligonucleotides were obtained by nanoprecipitation by adapting a previously reported protocol.^[^ ^52^ ^]^ A stock solution of PLGA (Resomer RG 752 H, Poly(D,L‐lactide‐*co*‐glycolide) in acetone with 1 mg mL^−1^ concentration was prepared. Then, a selected PLGA solution volume of 127 µL (as detailed in Figure S1, Supporting Information) was dropped into 1 mL of DE‐DOPE/miRNA lipoplex suspension, under magnetic stirring for 30 min to get hybrid NPs with 95% w/w PLGA content. Upon solvent removal under rotary evaporation, 1 mL of hybrid NPs suspension was obtained. Control PLGA NPs, not encapsulating miRNA‐loaded lipoplexes, were obtained through the nanoprecipitation of 127 µL PLGA solution with 1 mg mL^−1^ concentration in 1 mL water.

Scalability of the hybrid NPs preparation process was assessed by using both 2‐ and 8‐fold higher volumes of PLGA solution and DE‐DOPE/miRNA lipoplex suspension for nanoprecipitation. Upon acetone removal by evaporation, RNase‐free water was added to reach a constant final volume of 1 mL. Hybrid NPs with 2 and 8X concentrations with respect to the starting formulation (1X) were thus obtained (Table S4, Supporting Information).

Lipofectamine RNAiMAX (RNAiMAX, Invitrogen – Life Technologies) were prepared following manufacturer's instructions by diluting the transfection reagent and miRNA (3 µL 10 µm of negmiR, miR‐1, miRcombo) or Cy5‐siRNA in RNAse‐free water or culture medium depending on the following tests. The transfection reagent and the oligonucleotide were mixed and incubated for 5 min at room temperature.

### Physicochemical Characterization of Hybrid NPs—Characterization of Hybrid NPs and RNAiMAX

The hydrodynamic diameter, polydispersity index (PDI), and Z‐potential of hybrid NPs loaded with different oligonucleotides and control RNAiMAX were analyzed by dynamic light scattering (DLS) using a Litesizer 500 (Anton Paar). The Z‐potential of hybrid NPs and RNAiMAX was evaluated using a U‐shaped fold capillary cell (Anton Paar).

Hybrid NPs size and particles/mL concentration were measured through nanoparticle tracking analysis (NTA) using a NanoSight LM20 (NanoSight, Amesbury, U.K.). The samples were appropriately diluted and introduced into the sample chamber using sterile syringes. All measurements were carried out at room temperature in triplicate.

Oligonucleotide loading efficiency (LE) of hybrid NPs and RNAiMAX was evaluated by an indirect method by the measurement of free oligonucleotide concentration in water after NPs preparation. LE of RNAiMAX loaded with negmiR and hybrid NPs loaded with negmiR, miR‐1, Cy5‐siRNA, and miRcombo was analyzed. Briefly, hybrid NPs and RNAiMAX were centrifuged (15000 rpm, 15 min at 4 °C) using an Allegra X 30 benchtop centrifuge (Beckman Coulter). Supernatants were collected and analyzed by benchtop Qubit 4 Fluorometer assay following the manufacturer's instructions (MAN0009427‐Life Technologies). LE was calculated using the following equation, Equation (1), as the percentage ratio between the difference of the initial amount of miRNA used for NPs preparation and the quantity of free/non‐encapsulated miRNA in the supernatant after NPs preparation, and the initial miRNA used for NPs preparation:

(1) $$LE\;\left(\%\right)=\frac{Total\;miRNA\;-free\;miRNA\;in\;supernatant}{Total\;miRNA}\;\;\times 100$$

### Physicochemical Characterization of Hybrid NPs—Morphology

Morphology of negmiR‐loaded hybrid NPs was analyzed by cryogenic‐transmission electron microscopy (cryo‐TEM), using Philips CM120 microscope at the “Centre Technologique des Microstructures” (CTµ) at the Université Claude Bernard Lyon 1 (Villeurbanne, France). Diluted hybrid NPs suspension was dropped onto 300 Mesh holey carbon film (Quantifoil R2/1) and quench‐frozen in liquid ethane using a cryo‐plunge workstation (made at Laboratoire de Physique des Solides, LPS in Orsay). The specimens were then mounted on a precooled Gatan 62 specimen holder and observed at an accelerating voltage of 120 kV.

### Physicochemical Characterization of Hybrid NPs—Hybrid NPs Production Yield

The yield of hybrid NPs preparation process was calculated using Equation (2), as the percentage ratio between the hybrid NPs weight, measured after 24 h freeze‐drying the final NPs suspension, using Cool Safe 4–15 L Freeze Dryers (LaboGene, Lillerød, Denmark), and the sum weight of starting components. The hybrid NPs production yield was measured in triplicate.

(2) $$Yield\;\left(\%\right)=\frac{Final\;NPs\;weight\;\left(mg\right)}{Initial\;weight\;\left(mg\right)}\;\;\times 100$$

### Physicochemical Characterization of Hybrid NPs—Stability Study in Different Media

The physical stability of hybrid NPs loaded with negmiR or miRcombo was analyzed at 37 °C in RNase‐free water, phosphate buffered solution (PBS, Gibco) and Dulbecco's Modified Eagle's Medium High Glucose (DMEM, Gibco) supplemented with 10% of fetal bovine serum (FBS, Gibco) with a final volume of 1 mL. Samples were collected at predetermined time intervals (immediately after NPs formulation and at 4, 6, 24, and 48 h). Hydrodynamic size, PDI, and Z‐potential were measured by DLS analysis. All measurements were performed in triplicate.

### Physicochemical Characterization of Hybrid NPs—Evaluation of miRNA Release Kinetics In Vitro

The in vitro miRNA release kinetics was analyzed for hybrid NPs loaded with negmiR or miRcombo, incubated at 37 °C in RNase‐free water or 10 mm PBS with a final volume of 1 mL in dynamic conditions at different times (24, 48, 72, 168 and 192 h). At each time step, samples were centrifuged (14000 rpm, 15 min at 4 °C) using Fresco 21 Microcentrifuge (Life Technologies, Carlsbad, California, USA) and 950 µL of supernatant was collected. For the analysis, 10 µL of the supernatant were added to 190 µL of the working solution (obtained by diluting Qubit microRNA reagent in Qubit microRNA buffer 1:200), rapidly vortexed, and analyzed by Qubit 4 Fluorometer. The instrument provided the concentration (µg/mL) of free miRNA released in the supernatant at each time step. After the analysis, 950 µL of fresh RNase‐free water or PBS was added to each sample to replace the removed supernatant. Experiment was performed in triplicate. The miRNA release study was also performed with RNAiMAX as a comparison.

### In Vitro Cell Culture and Transfection

Adult Human Cardiac fibroblasts (AHCFs, CC‐2903, Lonza) were maintained in culture using Cardiac Fibroblasts Growth Medium‐3 (CC‐4526, Lonza) containing 10% FBS, 1% insulin, 1% human basal fibroblast growth factor (hFGF‐B) and 1% gentamicin. Cells were expanded until passage 4 and then used for the experiments. For all studies, AHCFs were seeded at 11.000 cells cm^−2^ in DMEM supplemented with 10% FBS and 1% L‐glutamine (Gibco). After 24 h, cells were transfected as described in next paragraphs depending on the experimental methods.

### In Vitro Uptake Efficiency of Hybrid NPs by Cells

Uptake efficiency of hybrid NPs by AHCFs was assessed using flow cytometry and fluorescence microscopy after 24 h of treatment. AHCFs were plated in 24‐well plate at 11.000 cells cm^−2^ density using 500 µL complete culture medium, or in µ‐Slide 18 well (Ibidi) using 100 µL complete culture medium for flow cytometry and confocal microscopy analyses, respectively. After 24 h, cells were treated with hybrid NPs loaded with Cy5‐siRNA for additional 24 h (at 25 nm final concentration).

For flow cytometry, non‐treated cells and cells treated with Cy5‐siRNA loaded RNAiMAX were also used as controls. AHCFs were harvested 24 and 48 h post treatments, washed twice with PBS, and detached with 0.25% w/v trypsin/ethylenediaminetetraacetic acid (EDTA, Gibco), centrifuged and resuspended in PBS. Flow cytometry was performed using Guava Easy Cyte flow cytometer (Cytek Biosciences B.V.) and analyzed using GuavaSoft 3.2 software (Cytek Biosciences B.V.) to obtain the percentage of cells positive for Cy5‐siRNA (Cy5‐siRNA^+^ AHCFs) and their Mean Fluorescence Intensity (M.F.I.). Experiments were performed in triplicate.

For fluorescence microscopy analysis, 24 h after treatment, cells were washed twice with PBS and fixed with paraformaldehyde 4% (Alfa Aesar) for 10 min at room temperature. Nuclei and cytoskeleton were counterstained with 4′,6‐diamidino‐2‐phenylindole dihydrochloride (DAPI, Life Technologies) and Alexa‐555 Phalloidin (Life Technologies) for 15 min at room temperature. Images were acquired using Nikon Eclipse Ti2 spinning disk microscope and NIS‐Elements software (Nikon). Merge of images for different color channels was performed using ImageJ (Fiji) software.

### Cytocompatibility and Cytotoxicity Assays

AHCFs were seeded at 11.000 cells cm^−2^ density on 96‐well plate in 100 µL of complete medium for 24 h. Then, AHCFs were treated with hybrid or RNAiMAX NPs loaded with negmiR for 24 h (100 µL). In the case of hybrid NPs, different concentrations of NPs suspension were tested by diluting the original formulation as detailed in Table 1. Non‐treated AHCFs were incubated with Lysis Solution (Promega) to induce cell cytotoxicity and considered as positive control for cytotoxicity analysis (100% cell toxicity). Cell medium was withdrawn after 24 h and placed in a black‐side clear bottom 96‐multiwell (Corning). CytoTox‐ONE (Promega) was added to each well following the manufacturer's instruction to evaluate cytotoxicity.

For cytocompatibility assay, cells treated with NPs for 24 h were washed with PBS and incubated with CellTiter‐Blue (Promega) in a complete medium for 3 h at 37 °C. Non‐treated AHCFs were also incubated with CellTiter‐Blue containing medium and used as positive control for cytocompatibility analysis (100% cell viability). Then, medium (from each well) was withdrawn and placed in a black‐side clear bottom 96‐multiwell (Corning).

For both cytotoxicity and cytocompatibility assays, samples were analyzed using a spectrophotometer Varioskan LUX Multimode Microplate Reader (Life Technologies, Carlsbad, California, USA) by setting excitation and emission wavelengths at 560 and 590 nm, respectively.

### Treatment of AHCFs with Hybrid NPs for the Assessment of In Vitro Transfection Efficiency and Direct Cell Reprogramming by Gene Expression Analysis

To study transfection efficiency by miRNA‐loaded NPs, AHCFs were plated in 24‐well plate (2.2  × 10^4^ cell well^−1^) in complete medium and cultured for 24 h. Then, cells were treated with hybrid NPs, loaded with miR‐1 or negmiR, in culture medium (500 µL volume, RNA concentration of 25 nm, 25 pmol). After 24 h, cells were washed twice with PBS and fresh complete medium was added. Culture was continued for additional 24 h before RNA extraction to analyze the expression of TWF‐1 mRNA as the target of miR‐1.

To preliminarily study AHCFs reprogramming into iCMs, AHCFs were treated with miRcombo or negmiR loaded hybrid NPs in complete medium volume (500 µL volume, with miRNA concentration of 25 nm, 25 pmol). After 24 h, cells were washed twice with PBS and fresh complete medium was added. Culture was continued for additional 15 days, before RNA extraction.

### RNA Expression and ddPCR Analysis

RNA was extracted using TRIzol (Invitrogen) lysis reagent, according to manufacturer's instructions. RNA quantity and quality were assessed using Synergy HTX microplate reader (Biotek). Total cDNA (200 ng) was obtained using High‐capacity cDNA reverse transcription kit (Applied Biosystems). Gene expression in AHCFs was examined by droplet digital PCR (ddPCR, Bio‐Rad Laboratories). Droplet generation was performed according to manufacturer's instructions. Thermal‐cycling conditions were 95 °C for 10 min (1 cycle), 94 °C for 30 s and 55 °C for 30 s (40 cycles), 98 °C for 10 min (1 cycle), and 4 °C infinite hold. In all ddPCR experiments, PCR plate was loaded on Bio‐Rad QX100 droplet reader for quantification of cDNA copies/mL. Analysis of the ddPCR data was performed by QuantaSoft analysis software (Bio‐Rad Laboratories). No template control with H_2_O was included in each assay. Experiments were performed in triplicate and repeated three times. Results were reported as concentration (cDNA copies/mL) of the gene of interest calculated on the concentration mean (cDNA copies/mL) of GAPDH.

### Assessment of In Vitro Direct Reprogramming Efficiency of AHCFs by Flow Cytometry

Flow cytometry was performed to evaluate in vitro direct reprogramming efficiency of AHCFs, transfected with negmiR or miRcombo loaded hybrid NPs for 24 h and then, cultured in complete culture medium for 15 days. Cells were treated with 0.05% trypsin/EDTA and permeabilized with 0.5% v/v Tween 20 (Sigma–Aldrich) in PBS for 5 min. Ice‐cold PBS with 10% FBS and 1% sodium azide (Sigma Aldrich) was used for washing between each step. Cells were incubated with Cardiac Troponin T (cTnT) primary antibody (cat #701620, Invitrogen) for 1 h at 4 °C and Alexa Fluor 488‐conjugated secondary antibody (ab150077, Abcam) for 1 h at 4 °C in the dark. Cells were run on Guava EasyCyte flow cytometer (Cytek Biosciences B.V) and data analysis was performed using GuavaSoft 3.2. The percentage of cTnT‐positive cells was calculated as an index of in vitro direct reprogramming efficiency.

### Stability of Physical and Functional Properties of Hybrid NPs Upon Storage

The physical stability of hybrid NPs, loaded with negmiR as negative control, miRcombo or with miR‐1 for in vitro transfection efficiency test, was evaluated at different storage conditions, such as: i) incubation of the hybrid NPs suspension at 4 °C; ii) freezing at −80 °C of the hybrid NPs suspension (1 mL) with/without cryoprotectant (trehalose 012250, ACEF) followed by storage at −80 °C; iii) freeze‐drying of the hybrid NPs suspension (1 mL) with trehalose followed by storage at −20 °C.

For storage condition (i), hybrid NPs suspensions were stored at 4 °C for different times before analyzing the maintenance of physical and functional properties, with respect to fresh NPs suspensions. Stability of physical properties of negmiR‐ and miRcombo‐loaded hybrid NPs stored at 4 °C was evaluated by measuring the hydrodynamic diameter, PDI, and Z‐potential after 7, 14, 21, and 28 days of storage, through DLS analysis, compared to freshly prepared NPs suspension. To assess the maintenance of functional properties during storage, in vitro cell tests were performed. AHCFs were treated with miR‐1 loaded hybrid NPs stored at 4 °C for 48 h, 7, and 14 days, and TWF‐1 downregulation was analyzed 48 h post‐transfection. RNA extraction and ddPCR analysis were performed as described in the above paragraphs.

For storage condition (ii), freshly prepared hybrid NPs suspensions were centrifuged (at 15000 rpm, for 15 min at 4 °C) using an Allegra X 30 benchtop centrifuge (Beckman Coulter) to remove the supernatant. Then, a water solution (1 mL) with different trehalose concentrations (0, 7, 10, 12% w/v) was added, before freezing at −80 °C. Frozen hybrid NPs were stored at −80 °C for 7 and 14 days. For storage condition (iii), freshly prepared hybrid NPs suspensions were centrifuged (at 15000 rpm, for 15 min at 4 °C) using an Allegra X 30 benchtop centrifuge to remove the supernatant. Then, a water solution (1 mL) with different trehalose concentrations (2.5, 5, 7, 10, 12, 13, 15, and 18% w/v) was added, before freezing at −80 °C for 2 h and subsequently freeze‐drying overnight using Cool Safe 4–15L Freeze Dryer (LaboGene, Lillerød, Denmark). Then, the lyophilized powder was stored at −20 °C for 7 and 14 days. For further analyses after storage conditions (ii) and (iii), frozen hybrid NPs were thawed, while freeze‐dried hybrid NPs were reconstituted in 1 mL RNase‐free water. After thawing or reconstitution, hybrid NPs were washed by means of a centrifugation step at 15000 rpm for 15 min at 4 °C and then resuspended in RNase‐free water. Stability of physical properties of negmiR‐loaded hybrid NPs after storage conditions (ii) and (iii) for 0, 7, and 14 days was evaluated by measuring the hydrodynamic diameter, PDI, and Z‐potential of NPs. Cell viability of AHCFs transfected for 24 h with negmiR‐loaded hybrid NPs after storage conditions (ii) and (iii) for 0, 7, and 14 days was evaluated as described in above paragraphs. For in vitro tests, AHCFs were treated with miR‐1 loaded hybrid NPs kept for 0, 7, and 14 days under storage conditions (ii) and (iii), and TWF‐1 downregulation was analyzed 48 h post‐transfection. RNA extraction and ddPCR were performed as described in previous paragraphs.

### Analysis of Microbial Contamination of Hybrid NPs During Storage

Analysis of microbial contamination of freshly prepared hybrid NPs and frozen and freeze‐dried hybrid NPs (in the presence of 12% w/v trehalose) after storage for 0, 7, and 14 days, respectively, at −80 and −20 °C was conducted through the direct inoculation method, following European Pharmacopeia (Ph. Eur. 2.6.1, 2008)^[^ ^74^ ^]^ and ISO 11737‐2:2019 Sterilization of health care products (Bioburden estimation). Hybrid NPs suspensions (200 µL, 26.7 µg mL^−1^) from each storage condition were incubated in 2.8 mL of trypcase‐soya broth (TSB, for aerobic bacteria; STBMTSB12, Merk) and thioglycolate broth (TGB, for anaerobic bacteria; STBMFTM12, Merk) at 25 and 37 °C, respectively. After 1, 7, and 14 days of incubation, the presence of bacterial contamination was investigated by visual observation of turbidity, by the colony‐forming unit (CFU) method on solid agar plates and by spectroscopy analysis. For the CFU method, 100 µL of hybrid NPs suspensions at each time point was spread onto a solid agar plate and incubated for 24 h at 25 °C for samples TSB and at 37 °C for TGB samples. For spectroscopy analysis, 100 µL of hybrid NPs suspensions at each time point were analyzed using Varioskan LUX Multimode Microplate Reader (Life Technologies, Carlsbad, California, USA). Absorbance spectrum was acquired between 400 and 700 nm. Single absorbance wavelengths (457, 490, and, 600 nm), which were specific wavelengths for possible microbial contamination, were analyzed. All the analyses were performed in triplicate. Each sample was compared to the negative control.

### Statistical Analysis

All measurements were performed in triplicate. Physicochemical characterization data are presented as mean values ± standard deviation (SD). Statistical analysis was performed using One‐way Anova tests and Student's *t*‐test. For biological experiments, the results were presented as means ± standard error of the mean (SEM) of triplicate experiments. Statistical analyses were performed with Student's *t*‐test. *P*‐value was reported as ^*^ *p* < 0.05 considered statistically significant, ^**^ *p* < 0.01 considered highly significant, and ^***^ *p* < 0.0001 very highly significant. All graphs were prepared using GraphPad software.

## Conflict of Interest

The authors declare no conflict of interest.

## Author Contributions

L.N, C.P, M.C, E.M, G.P.S, C.M, and V.C designed the experimental part and analyzed the data. L.N, M.C, and C.M conducted formulation and characterization of hybrid NPs, supported by I.A, F.C, and F.S. L.N, C.P, M.C, and E.M conducted storage stability studies and microbial contamination analysis of hybrid NPs. C.P., L.N., and M.C. designed the in vitro culture experiments and ddPCR analysis. C.P. conducted ddPCR analysis for hybrid NPs cytocompatibility, miR‐1 overexpression, and miRNA target downregulation. C.P., L.N., M.C., and C.M. conducted and supervised the in vitro culture experiments with adult human cardiac fibroblasts. V.C., C.M., B.S., and S.A. supervised the work. V.C. acquired funding and supervised the project. The manuscript was written through main contributions by L.N, C.P, M.C, E.M, C.M, and V.C and further help by all the authors. All authors have given approval to the final version of the manuscript.

## Supporting information



Supporting Information

## Data Availability

The data that support the findings of this study are available on request from the corresponding author. The data are not publicly available due to privacy or ethical restrictions.
