# Supplementary material for: Design and Validation of Hybrid Polymer‐Lipid Nanoparticles as Novel Transfection Vectors for MicroRNA Delivery to Human Cardiac Fibroblasts
Source: Adv Healthc Mater. 2025 Jun 6;14(18):2500971. doi: 10.1002/adhm.202500971 (PMC12264849; doi:10.1002/adhm.202500971)
Supplement: Supplementary file 1 — Supporting Information [file ADHM-14-0-s001.docx]

**Supplementary information**

**Design and validation of hybrid polymer-lipid nanoparticles as novel transfection vectors for microRNA delivery to human cardiac fibroblasts.**

*Letizia Nicoletti*^1,2^, Camilla Paoletti^1^, Martina Coletto^1^, Elena Marcello^1^, Giovanni Paolo Stola^1,2^, Francesca Cossetta^1^, Francesco Schiavone^1^, Ilaria Andreana^3^, Barbara Stella^3^, Silvia Arpicco^3^, Clara Mattu ^1^ and Valeria Chiono^1,2^*

^1^ Department of Mechanical and Aerospace Engineering, Politecnico di Torino, Corso Duca degli Abruzzi 24, 10129 Turin, Italy

^2^ PoliRNA Srl, Via Vincenzo Vela 42, 10128 Turin, Italy.

^3^ Department of Drug Science and Technology, University of Turin, Via Pietro Giuria, 9, 10125, Turin, Italy

**Optimization of PLGA amount in hybrid NPs.**

Hybrid NPs loaded with oligonucleotides were obtained by nanoprecipitation by adapting a previously reported protocol. A stock solution of PLGA (Resomer^®^ RG 752 S, Poly(D,L-lactide-*co*-glycolide) in acetone with 1 mg/mL concentration was prepared. Then, different amounts of PLGA solution (16, 20, 24, 27, 38, 60 and 127 µg) were dropped into 1 mL of DE-DOPE/miRNA lipoplex suspension (N/P ratio 3 containing 6 µg DE-DOPE and 0.7 µg miRNA, corresponding to 50 pmol miRNA), under magnetic stirring for 30 min, corresponding to 70, 75, 78, 80, 85, 90 and 95 % w/w of PLGA in the resulting hybrid NPs. Upon solvent removal under rotary evaporation, 1 mL of hybrid NPs suspension was obtained. Based on the analysis of the hydrodynamic diameter, PDI and Z-potential, hybrid NPs with 95 % w/w PLGA were selected (Figure S1 and Table S1).


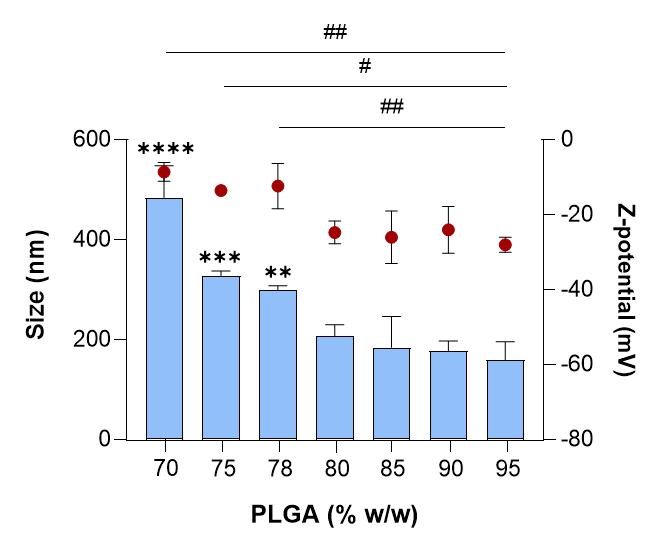


**Figure S1:** Hydrodynamic diameter and Z-potential of hybrid NPs with different PLGA contents (70, 75, 78, 80, 85, 90 and 95 % w/w). Data are expressed as mean ± SD. Statistical analysis was performed by 1-way ANOVA.


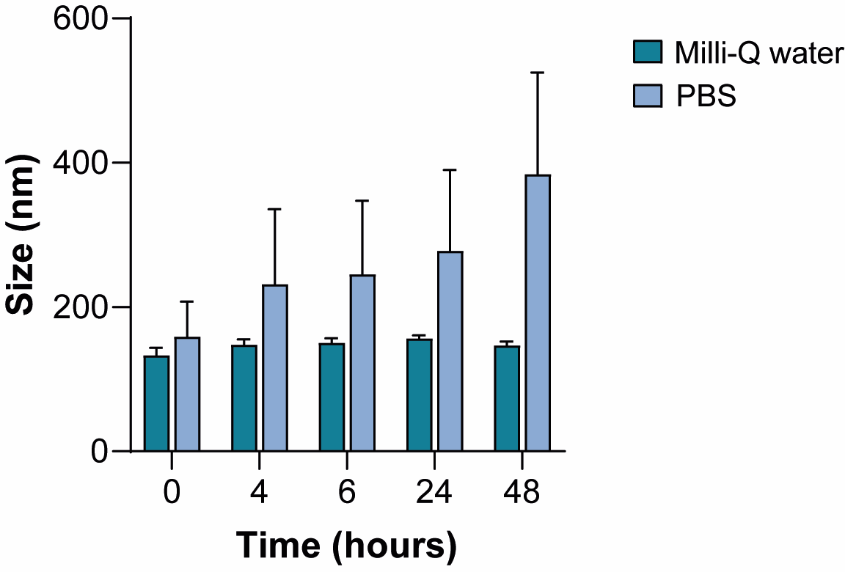


**Figure S2:** Stability study at 37 °C in Milli-Q water and in PBS by measuring hydrodynamic diameter of hybrid NPs as a function of their incubation time (0 h, 4 h, 6 h, 24 h and 48 h). Data are expressed as mean ± SD of three independent experiments. Statistical analysis was performed by 1-way ANOVA.

**Endosome/Lysosome staining for Cy5-siRNA localization**

Endosome/lysosome escape mediated by hybrid NPs in AHCFs was assessed using microscopy after 24 h of treatment. AHCFs were plated in µ-Slide 18 well (Ibidi) using 100 µL complete culture medium. After 24 h, cells were treated with hybrid NPs and RNAiMAX NPs loaded with Cy5-siRNA for additional 24 h (at 25 nM final concentration). Then, cells were washed twice with PBS and CytoPainter Lysosomal Staining Kit (Abcam – ab112136) was used to counterstain endosome/lysosome following manufacturer’s instructions. Images were acquired using Nikon Eclipse Ti2 spinning disk microscope and NIS-Elements software (Nikon). Merge of images for different colour channels was performed using ImageJ (Fiji) software. Pearson's correlation coefficient (PCC) between the fluorescent signal of Cy5-siRNA loaded RNAiMAX NPs and hybrid NPs and lysosomes. PCC analysis was performed by using ImageJ software.

**
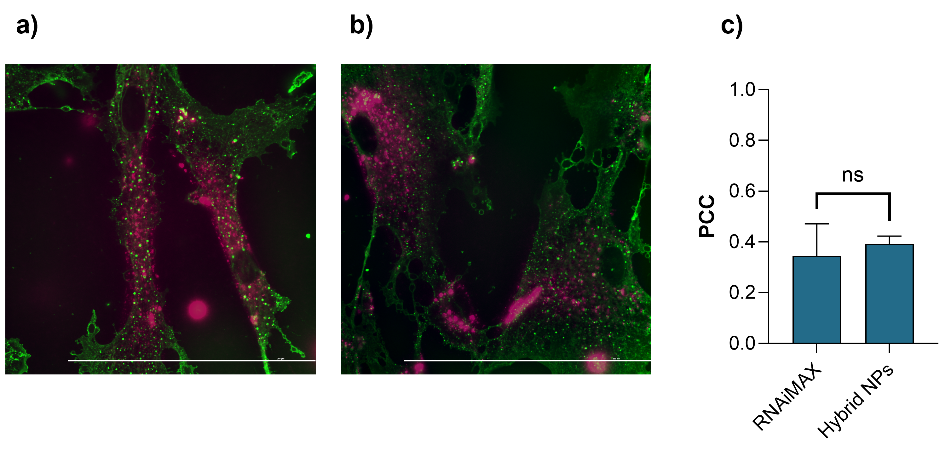
**

**Figure S3.1:** Fluorescence microscopy image showing Cy5-siRNA (magenta) loaded in a) RNAiMAX NPs and b) hybrid NPs and lysosomes (green) after 24 h treatment. Scale bar = 250 µm. c) Pearson's correlation coefficient (PCC) between the fluorescent signal of Cy5-siRNA loaded RNAiMAX NPs and hybrid NPs and lysosomes. Data are expressed as mean ± SEM of three independent experiments. Statistical analysis was performed by two-sided t-test. PCC analysis was performed by using ImageJ software.

**
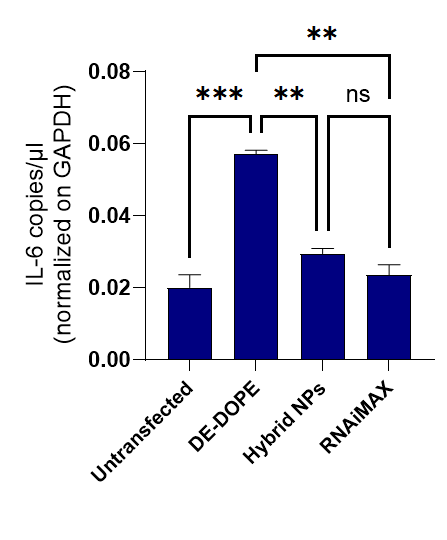
**

**Figure S3.2:** Gene expression of IL-6 mRNA in AHCFs transfected with DE-DOPE, hybrid NPs and RNAiMAX loaded with negmiR for 24 h, analysed by ddPCR. Results are reported as number of mRNA copies/µl and normalized on GAPDH expression. Untransfected cells were used as control. Data are expressed as mean ± SEM of three independent experiments. Statistical analysis was performed by two-sided t-test.


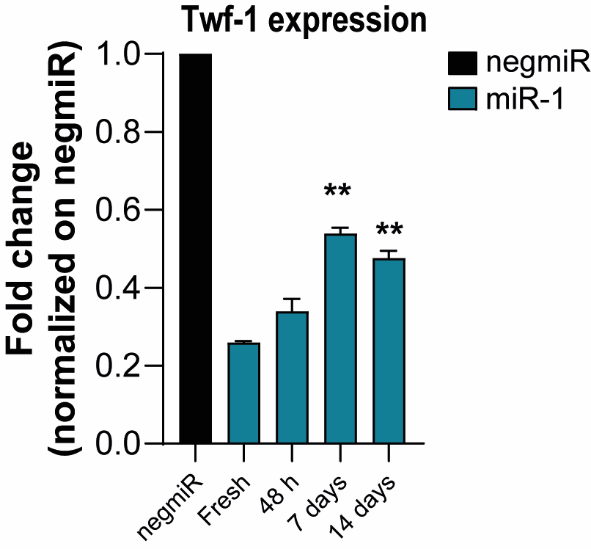


**Figure S3.3:** Expression of TWF-1 mRNA target in AHCFs transfected with negmiR or miR-1 using hybrid NPs freshly prepared or stored in suspension at 4 °C for 48 h, 7 days and 14 days. TWF-1 mRNA expression was analysed at 48 h post-transfection by ddPCR. Data are reported as fold change expression compared to negmiR controls. Data are expressed as mean ± SEM of three independent experiments. Statistical analysis was performed by two-sided t-test.


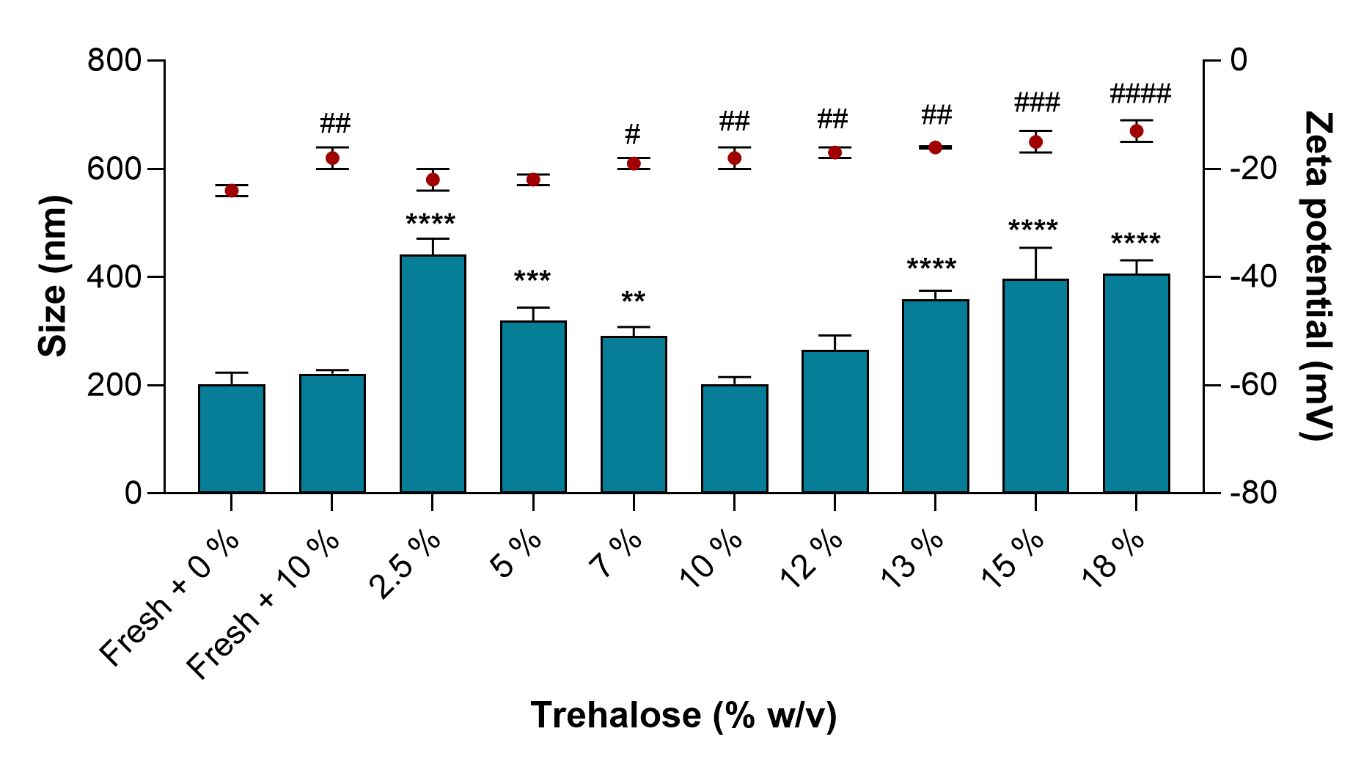


**Figure S4:** Hydrodynamic diameter and Z-potential of hybrid NP suspensions with different concentrations of trehalose (2.5, 5, 7, 10, 12, 13, 15, and 18 % w/v), subjected to freeze-drying followed by resuspension, compared to freshly prepared hybrid NPs (0 % and 10 % w/v trehalose). NegmiR was loaded as model miRNA. Data are expressed as mean ± SD. Statistical analysis was performed by two-sided t-test.

**
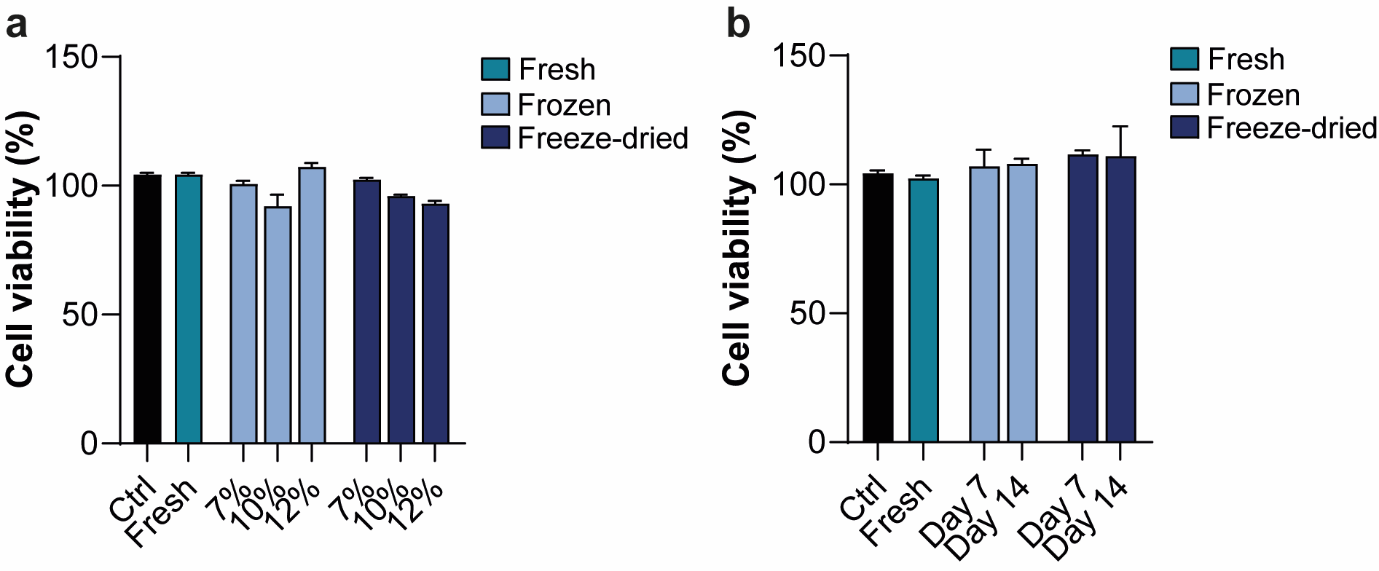
**

**Figure S5: (a)** Viability of AHCFs transfected for 24 h with different batches of hybrid NPs: freshly prepared with no trehalose; frozen or freeze-dried with different concentrations of trehalose (7, 10, and 12 % w/v) and resuspended. Viability of transfected AHCFs was normalized to non-transfected AHCFs (Ctrl). **(b)** Viability of AHCFs transfected for 24 h with hybrid NPs frozen or freeze-dried with 12 % w/v trehalose, stored for 7 and 14 days at -20°C and then resuspended, compared to treatment with fresh hybrid NPs with no trehalose. Viability of transfected AHCFs was normalized to non-transfected AHCFs (Ctrl). Data are expressed as mean ± SEM of three independent experiments. Statistical analysis was performed by two-sided t-test.

**Table S1** Hydrodynamic diameter, PDI and Z-potential of hybrid NPs with different PLGA contents (70, 75, 78, 80, 85, 90 and 95 % w/w). Data are expressed as mean ± SD.

| **% PLGA**  **(w/w)** | **Size**  **(d. nm)** | **PDI** | **Z-potential**  **(mV)** |
| --- | --- | --- | --- |
| **70** | 449 ± 30 | 0.45 ± 0.05 | - 12 ± 5 |
| **75** | 328 ± 9 | 0.28 ± 0.11 | -17 ± 7 |
| **78** | 299 ± 9 | 0.23 ± 0.03 | -12 ± 6 |
| **80** | 208 ± 22 | 0.26 ± 0.02 | -24 ± 3 |
| **85** | 185 ± 62 | 0.26 ± 0.03 | -26 ± 7 |
| **90** | 178 ± 22 | 0.28 ± 0.01 | -26 ± 7 |
| **95^a^** | 160 ± 36 | 0.27 ± 0.02 | -28 ± 2 |

^a^ selected composition, which from this point on is referred in the text as hybrid NPs.

**Table S2** Hydrodynamic diameter, PDI, and Z-potential of control unloaded PLGA NPs (non-encapsulating miRNA) and RNAiMAX NPs (loaded with negmiR and prepared following manufacturer’s instructions), measured by DLS analysis. Data are expressed as mean ± SD.

| **Samples** | **Size**  **(d. nm)** | **PDI** | **Z-potential**  **(mV)** |
| --- | --- | --- | --- |
| **PLGA NPs** | 59 ± 1 | 0.11 ± 0.02 | -20 ± 2 |
| **RNAiMAX NPs** | 806 ± 37 | 0.34 ± 0.02 | 56 ± 2 |

**Table S3** PDI and Z-potential of hybrid NPs loaded with different oligonucleotides (Cy5-siRNA, miR-1 and miRcombo) measured by DLS analysis. Data are expressed as mean ± SD.

| **Oligonucleotides** | **PDI** | **Z-potential**  **(mV)** |
| --- | --- | --- |
| **Cy5-siRNA** | 0.26 ± 0.01 | -37 ± 2 |
| **miR-1** | 0.29 ± 0.01 | -26 ± 3 |
| **miRcombo** | 0.29 ± 0.02 | -41 ± 2 |

**Table S4** Concentration (µg/mL), final volume (mL), miRNA concentration (nM) and miRNA amount (nmol) of hybrid NP suspensions prepared from 1X, 2X and 8X volumes of PLGA solution and DE-DOPE/miRNA lipoplex suspension; loading efficiency (LE%) and PDI of resulting hybrid NPs. Data are expressed as mean ± SD.

| **Code** | **Hybrid NP suspension concentration**  **(µg/mL)** | **Volume of suspension**  **(mL)** | **miRNA concentration**  **(nM)** | **miRNA amount**  **(nmol)** | **LE%** | **PDI** |
| --- | --- | --- | --- | --- | --- | --- |
| **1X** | 133.7 | 1 mL | 50 | 0.05 | 99 ± 0.2 | 0.27 ± 0.02 |
| **2X** | 267.4 | 1 mL | 100 | 0.10 | 99 ± 0.3 | 0.25 ± 0.06 |
| **8X** | 1069.6 | 1 mL | 400 | 0.40 | 99 ± 0.1 | 0.25 ± 0.01 |

**Table S5** Z-potential of hybrid NPs incubated in various media at 37 °C for different times. Data are expressed as mean ± SD.

| **Media** | **Z-potential**  **(mV)** | | | | |
| --- | --- | --- | --- | --- | --- |
|  | **0 h** | **4 h** | **6 h** | **24 h** | **48 h** |
| **milliQ** | -24 ± 5 | -25 ± 4 | -24 ± 3 | -26 ± 0.3 | -23 ± 2 |
| **PBS** | -31 ± 3 | -40 ± 4 | -39 ± 4 | -35 ± 5 | -32 ± 9 |
| **DMEM + FBS** | -14 ± 0.3 | -15 ± 1 | -14 ± 1 | -14 ± 2 | -10 ± 8 |

**Table S6** PDI of hybrid NPs, loaded with negmiR or miRcombo, stored at 4 °C in RNase free water. Data are expressed as mean ± SD.

| **miRNA cargo** | **PDI** | | | | |
| --- | --- | --- | --- | --- | --- |
|  | **Day 0** | **Day 7** | **Day 14** | **Day 21** | **Day 28** |
| **negmiR** | 0.27 ± 0.05 | 0.26 ± 0.03 | 0.21 ± 0.06 | 0.28 ± 0.01 | 0.24 ± 0.02 |
| **miRcombo** | 0.29 ± 0.02 | 0.30 ± 0.01 | 0.28 ± 0.02 | 0.26 ± 0.02 | 0.31 ± 0.02 |

**Table S7** PDI of freshly prepared versus frozen and resuspended hybrid NPs with no trehalose. Data are expressed as mean ± SD.

| **Hybrid NP Samples** | **PDI** |
| --- | --- |
| **Fresh** | 0.21 ± 0.02 |
| **Frozen** | 0.24 ± 0.04 |

**Table S8** PDI of freshly prepared versus freeze-dried and resuspended hybrid NPs with different concentrations of trehalose. Data are expressed as mean ± SD.

| **Trehalose (% w/v)** | **PDI** |
| --- | --- |
| **0 (Fresh)** | 0.25 ± 0.06 |
| **10 (Fresh)** | 0.27 ± 0.01 |
| **2.5** | 0.30 ± 0.01 |
| **5** | 0.26 ± 0.01 |
| **7** | 0.22 ± 0.04 |
| **10** | 0.24 ± 0.02 |
| **12** | 0.21 ± 0.03 |
| **13** | 0.26 ± 0.02 |
| **15** | 0.25 ± 0.01 |
| **18** | 0.23 ± 0.06 |

**Table S9** PDI of hybrid NPs frozen with different concentrations of trehalose and reconstituted. Data are expressed as mean ± SD.

| **Trehalose (% w/v)** | **PDI** |
| --- | --- |
| **7** | 0.28 ± 0.02 |
| **10** | 0.26 ± 0.01 |
| **12** | 0.27 ± 0.02 |

**Table S10** PDI of hybrid NPs, frozen or freeze-dried with 12 % w/v of trehalose and then stored for 7 and 14 days before resuspension. Data are expressed as mean ± SD.

| **Storage time** | **PDI** | |
| --- | --- | --- |
|  | **Frozen** | **Freeze-dried** |
| **Day 0** | 0.27 ± 0.02 | 0.21 ± 0.03 |
| **Day 7** | 0.26 ± 0.04 | 0.28 ± 0.03 |
| **Day 14** | 0.26 ± 0.01 | 0.30 ± 0.02 |
